# The Indian cobra reference genome and transcriptome enables comprehensive identification of venom toxins

**DOI:** 10.1038/s41588-019-0559-8

**Published:** 2020-01-06

**Authors:** Kushal Suryamohan, Sajesh P. Krishnankutty, Joseph Guillory, Matthew Jevit, Markus S. Schröder, Meng Wu, Boney Kuriakose, Oommen K. Mathew, Rajadurai C. Perumal, Ivan Koludarov, Leonard D. Goldstein, Kate Senger, Mandumpala Davis Dixon, Dinesh Velayutham, Derek Vargas, Subhra Chaudhuri, Megha Muraleedharan, Ridhi Goel, Ying-Jiun J. Chen, Aakrosh Ratan, Peter Liu, Brendan Faherty, Guillermo de la Rosa, Hiroki Shibata, Miriam Baca, Meredith Sagolla, James Ziai, Gus A. Wright, Domagoj Vucic, Sangeetha Mohan, Aju Antony, Jeremy Stinson, Donald S. Kirkpatrick, Rami N. Hannoush, Steffen Durinck, Zora Modrusan, Eric W. Stawiski, Kristen Wiley, Terje Raudsepp, R. Manjunatha Kini, Arun Zachariah, Somasekar Seshagiri

**Affiliations:** 1 Molecular Biology Department, Genentech, Inc., South San Francisco, CA USA; 2 MedGenome Inc., Foster City, CA USA; 3 AgriGenome Labs Private Ltd, Kochi, India; 4 SciGenom Research Foundation, Bangalore, India; 5 Molecular Cytogenetics laboratory, Texas A&M University, College Station, TX USA; 6 Ecology and Evolution Unit, Okinawa Institute of Science and Technology, Onna-son, Japan; 7 Department of Bioinformatics and Computational Biology, Genentech, Inc., South San Francisco, CA USA; 8 Center for Public Health Genomics, University of Virginia, Charlottesville, VA USA; 9 Department of Microchemistry Proteomics, and Lipidomics, Genentech, Inc., South San Francisco, CA USA; 10 The Donnelly Centre for Cellular and Biomolecular Research, University of Toronto, Toronto, Ontario Canada; 11 Division of Genomics, Medical Institute of Bioregulation, Kyushu University, Fukuouka, Japan; 12 Department of Pathology, Genentech, Inc., South San Francisco, CA USA; 13 College of Veterinary Medicine, Flow Cytometry Shared Resource Laboratory, Texas A&M University, College Station, TX USA; 14 Department of Early Discovery Biochemistry, Genentech, Inc., South San Francisco, CA USA; 15 Department of Molecular Biology, SciGenom Labs, Kochi, India; 16 Kentucky Reptile Zoo, Slade, KY USA; 17 Department of Biological Sciences, National University of Singapore, Singapore, Singapore; 18 Wayanad Wildlife Sanctuary, Sultan Bathery, India

**Keywords:** Sequence annotation, Genomics, Transcriptomics, DNA sequencing, RNA sequencing

## Abstract

Snakebite envenoming is a serious and neglected tropical disease that kills ~100,000 people annually. High-quality, genome-enabled comprehensive characterization of toxin genes will facilitate development of effective humanized recombinant antivenom. We report a de novo near-chromosomal genome assembly of *Naja naja*, the Indian cobra, a highly venomous, medically important snake. Our assembly has a scaffold N50 of 223.35 Mb, with 19 scaffolds containing 95% of the genome. Of the 23,248 predicted protein-coding genes, 12,346 venom-gland-expressed genes constitute the ‘venom-ome’ and this included 139 genes from 33 toxin families. Among the 139 toxin genes were 19 ‘venom-ome-specific toxins’ (VSTs) that showed venom-gland-specific expression, and these probably encode the minimal core venom effector proteins. Synthetic venom reconstituted through recombinant VST expression will aid in the rapid development of safe and effective synthetic antivenom. Additionally, our genome could serve as a reference for snake genomes, support evolutionary studies and enable venom-driven drug discovery.

## Abstract (web summary)

Analysis of a near-chromosomal genome assembly and transcriptome profiling of the Indian cobra identifies genes expressed in the venom glands. These data should help develop a new antivenom.

## Main

Fossil remains from ~100 million years ago (Ma) show that snakes were widely distributed across the world by the late Cretaceous period^1^. During the course of their evolution, snakes lost their limbs, acquiring a serpentine body^2^. Some also evolved or co-opted venom systems to help subdue, capture and digest their prey^2,3^. The Colubroides clade of advanced snakes encompasses >3,000 extant species including >600 venomous species^4^. The most venomous snakes include the true vipers and pit vipers, both members of the Viperidae family, and cobras, kraits, mambas and sea snakes from the Elapidae family^5^.

Although humans are not an intended target, accidental contact with venomous snakes can be deadly. Snakebite envenoming is a serious neglected tropical disease that affects ~5 million people worldwide annually, leading to ~400,000 amputations and >100,000 deaths^6^. In India alone, the high rural population density combined with the presence of the ‘big four’ deadly snakes, namely the Indian cobra (*Naja naja*), Russell’s viper (*Daboia russelli*), saw-scaled viper (*Echis carinatus*) and common krait (*Bungarus caeruleus*), results in >46,000 snakebite-related deaths annually^7^.

Snake venom is a potent lethal cocktail rich in proteins and peptides, secreted by specialized venom gland cells. Venom components can be broadly classified as neurotoxic, cytotoxic, cardiotoxic or hemotoxic, and the composition can vary both between and within species^8–11^.

Currently, snake antivenom is the only treatment effective in the prevention or reversal of the effects of envenomation. Since 1896, antivenom has been developed by immunization of large mammals, such as the horse, with snake venom to generate a cocktail of antibodies that are used for therapy^12^. Given the heterologous nature of these antibodies, they often elicit adverse immunological responses when treating snakebite victims^13^. Moreover, the antivenom composition is not well defined and its ability to neutralize the venom components is poorly understood. This is further exacerbated by the lack of access to antivenom and its high cost in many developing countries^14^. Although several alternative approaches have been proposed, large animal-based antivenom production using extracted snake venom as the antigen continues to be the standard practice^15–18^.

High-quality genomes of venomous snakes, combined with transcriptomics, will enable generation of a comprehensive catalog of venom-gland-specific toxin genes that can be used for the development of synthetic antivenom of defined composition using recombinant technologies. Thus far, only a few snake genomes have been published. A majority of these were generated primarily using short-read sequencing resulting in highly fragmented assemblies, thus limiting their utility for creating a complete catalog of venom-relevant toxin genes^19–25^. The ‘big four’ medically important snakes from India, including *N. naja*, are no exception and have not been well characterized at either the genome or transcriptome level. Only nine *N. naja* toxin gene sequences and 38 toxins, some of which probably represent the same gene, have been reported using mass spectrometry^26^.

In this study, using a number of genomic technologies, we have generated a de novo near-chromosome level reference genome assembly of *N. naja*, the Indian cobra. This high-quality genome allowed us to study various aspects of snake venom biology, including venom gene genomic organization, genetic variability, evolution and expression of key venom genes. Our integrated genome–transcriptome analysis identified a minimal set of 19 VST genes that constitute the core venom toxins. Targeting these core toxins should lead to the development of a safe and effective humanized antivenom.

## Results

### Near-chromosomal de novo genome assembly

**Extended Data Fig. 1:**
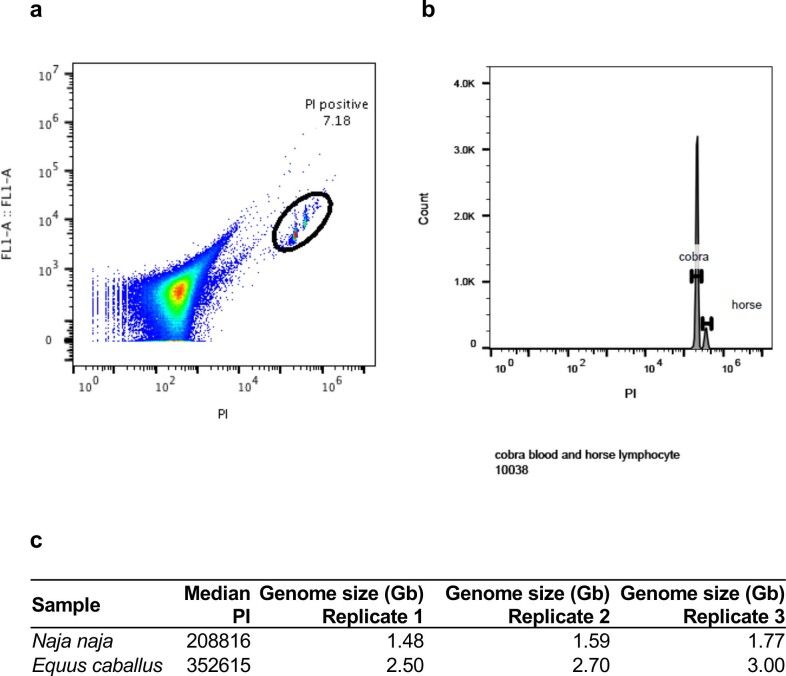
Indian cobra genome size estimation by flow cytometry. **a**, Gating strategy for Indian cobra (*Naja naja)* genome size estimation showing the propidium iodide (PI) positive sample within the elliptical gate. **b**, PI positive gated population in a histogram showing showing PI stained *N. naja* blood and *Equus caballus* (horse) lymphocytes. **c**, Table of median fluorescence intensities measured for *Naja naja* and *Equus caballus* and estimated genome size in Gb in 3 replicate experiments. A total of 3000 and 300 measurements were conducted for the Indian cobra and horse, respectively.

**Extended Data Fig. 2:**
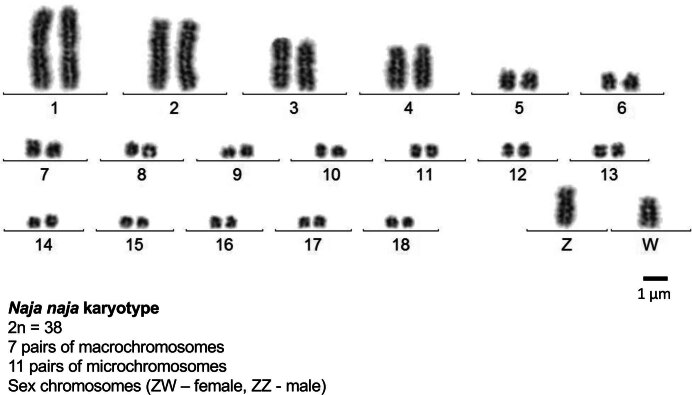
*N. naja* karyotyping. Representative karyotype obtained from cultured red blood cells from a female animal NN03. A total of N = 15 cells were karyotyped.

Using flow cytometry, we estimated the size of the Indian cobra haploid genome at 1.48–1.77 Gb (Extended Data Fig. 1). Cytogenetic analysis revealed a diploid karyotype of 2*n* *=* 38, comprising seven pairs of macrochromosomes (MACs), one pair of sex chromosomes (ZZ male or ZW female) and 11 pairs of microchromosomes (MICs), consistent with a previous report^27^ (Extended Data Fig. 2).

**Fig. 1:**
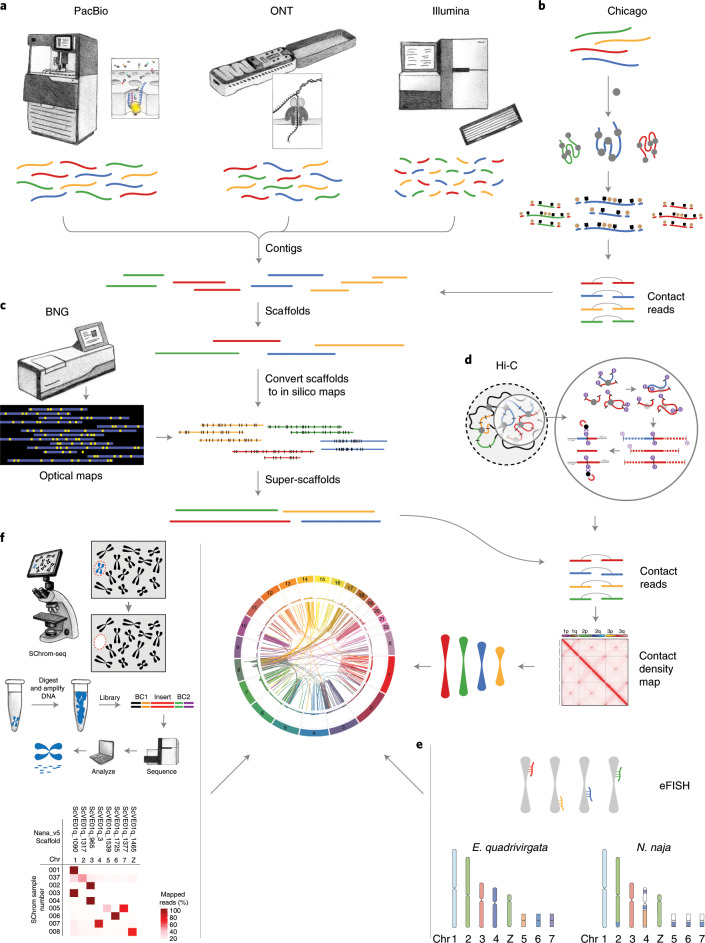
Schematic of *N. naja* genome sequencing and assembly. **a**,**b**, Long-read (PacBio and ONT) and short-read (lllumina) data (**a**) were used to build contigs that were then combined with Chicago^28^ chromatin interaction data (**b**) to generate scaffolds. **c**, Scaffolds from BNG optical mapping de novo assembly were combined with those from the previous step to generate super-scaffolds. **d**, Hi-C sequencing data were used to refine the assembly. **e**, Electronic fluorescence in situ hybridization (eFISH) was performed using cDNA FISH marker sequences from the Japanese rat snake, *E. quadrivirgata*. **f**, SChrom-seq data were used to assign scaffolds to chromosomes.

DNA from an adult male Indian cobra (NN01) was used to generate long-read (PacBio and Oxford Nanopore Technologies (ONT)), short-read (Illumina), Chicago^28^, Hi-C^29^ and optical mapping (Bionano Genomics (BNG)) data (Supplementary Table 1a,b). Additionally, we generated linked-read 10x Genomics, BNG and short-read Illumina sequence data for a female animal (NN05; Supplementary Note and Supplementary Table 1a,b). Our sequential assembly approach (Fig. 1 and Supplementary Note) resulted in a 1.79-Gb Nana_v5 genome with a scaffold N50 of 223.35 Mb and a BUSCO^30^ genome completeness score of 94.3% (Table 1 and Supplementary Table 2a–c).

**Table 1:** *N. naja* assembly summary statistics

Assembly	Contigs (*n*)	Scaffolds (*n*)	Gaps (Gb, %)	Contig N50 (Mb)	Scaffold N50 (Mb)	Assembly size (Gb)
Nana_v1 (long-read (LR))	13,066	–	–	0.31	–	1.66
Nana_v2 (LR + Chicago)	13,066	2,676	0.01 (0.6)	0.31	4.90	1.67
Nana_v3 (optical map (OM))	–	1,477	–	–	16.30	1.63
Nana_v4 (v3 + OM)	13,066	2,167	0.11 (6.4)	0.31	157.50	1.79
Nana_v5 (v4 + Hi-C)	13,066	1,897	0.11 (6.4)	0.30	223.35	1.79

To assign scaffolds from Nana_v5 to chromosomes, we used complementary DNA (cDNA) chromosome marker sequences from a colubrid, the Japanese rat snake *Elaphe quadrivirgata*^31^ (Fig. 1e), synteny information from *Anolis carolinensis* (the green anole lizard genome^32^) and single-chromosome sequencing data (SChrom-seq; Fig. 1f, Supplementary Table 3a–c and Supplementary Note). We also generated a hybrid 10x BNG genome assembly to identify a 52.1-Mb female-specific W chromosome-linked scaffold (Supplementary Table 3d and Supplementary Note).

Comparison of the *N. naja* de novo genome assembly to the human^33^ and goat^34^ genomes showed that the scaffold N50 of the *N. naja* genome was 2.5× (87.27 versus 223.35 Mb) and 3.3× (67.79 versus 223.35 Mb) greater than the goat and human genomes previously assembled using the involved, expensive, time-consuming physical and cytogenetic maps developed over a decade or more (Table 2). Compared with the king cobra genome^19^, also an elapid, the Indian cobra genome contained far fewer scaffolds (296,399 versus 1,897, respectively), and had 929-fold better contiguity (scaffold N50 of 0.24 versus 223.35 Mb, respectively). Also, compared to the 7,034 scaffolds and 179.89-Mb scaffold N50 reported recently for the prairie rattlesnake genome (*Crotalus viridis*)^35^, the Indian cobra genome had a higher scaffold N50 and fewer scaffolds (Table 2).

**Table 2:** Comparison of Nana_v5 assembly to other high-quality genomes

Assembly	Human GRCh38	Goat ARS1	King cobra	Five-pace viper	Indian cobra
Total sequence length (Gb)	3.2	2.9	1.66	1.47	1.79
Total assembly gap length (Mb)	160	38	210	82	110
Number of scaffolds	735	29,907	296,399	160,256	1,897
Number of scaffolds >10 Mb (% of assembly)	23 (99.8)	31 (90.1)	35 (2.7)	412 (72.0)	19 (94.9)
Scaffold N50 (Mb)	67.79	87.27	0.24	2.12	223.35
Number of chromosomes	23	31	18	18	19

### Genome features

**Fig. 2:**
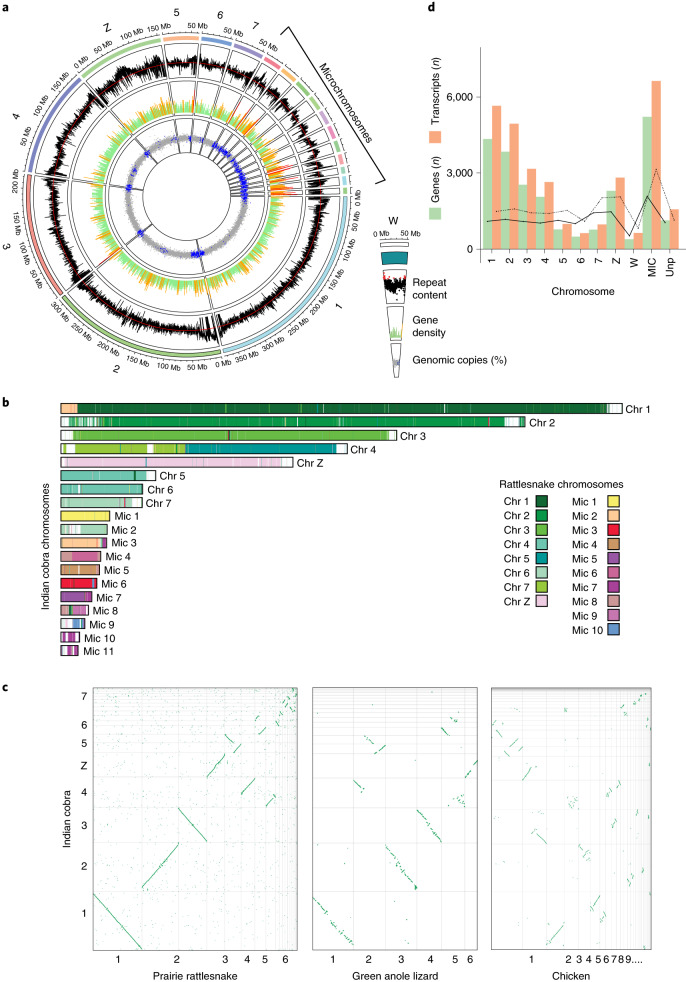
Genome architecture of *N. naja* genome. **a**, Circos plot of the reference genome assembly (NN01, a male adult (*n* = 1)) representing *N. naja* chromosomes (outermost track) from the Nana_v5 assembly, repeat content, gene density and GC content (%). Regions of the genome with GC content higher than average (40.46%) are shown in blue. Regions within the gene density of more than 10 genes are shown as red spikes, while those with 5 to 10 genes are indicated by yellow spikes. Green spikes represent regions with fewer than five genes. The average repeat content is indicated by the red line. All data were plotted in 100-kb windows. The female-specific W-linked scaffold obtained using NN05 DNA is shown on the right. **b**, Chromosome painting depicting synteny between Indian cobra and rattlesnake genomes. **c**, Dot-plots showing synteny of the Indian cobra genome with the prairie rattlesnake, chicken or green anole lizard genomes. **d**, Bar plot of the number of predicted genes and corresponding transcripts observed in Nana_v5. Dashed and solid lines denote average number of genes and transcripts detected in each chromosome along 100-Mb windows, respectively. MICs were combined into one group. Unp, unplaced scaffolds (*n* = 1,878) containing predicted genes.

**Extended Data Fig. 3:**
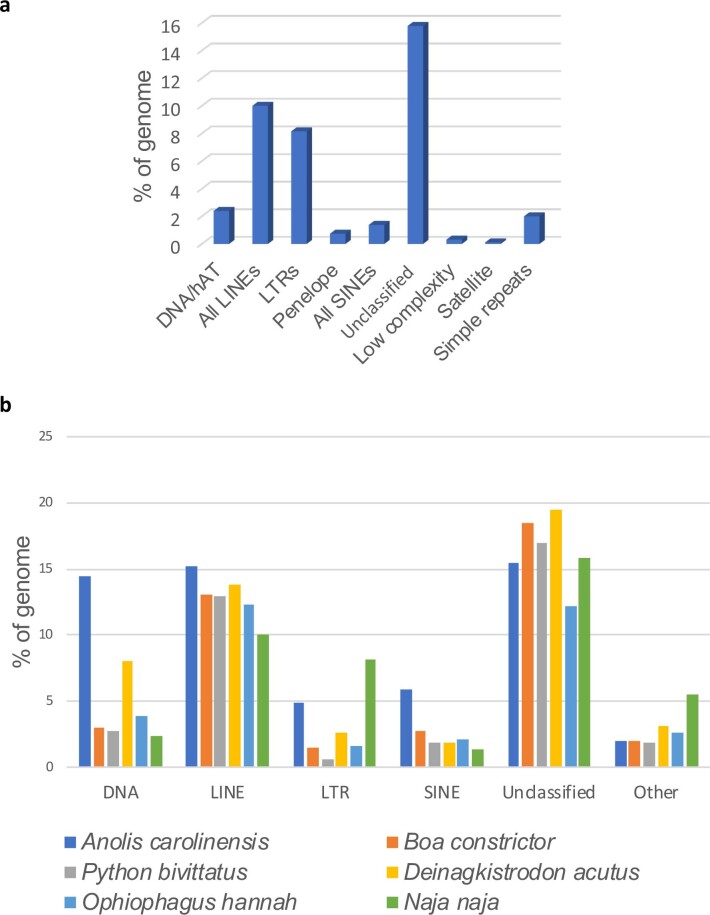
Genomic repeat elements identified in the Indian cobra genome. **a**, Bar plot of the percent distribution of the different classes of repeat elements in the *N. naja* genome (Nana_v5). **b**, Comparison of proportion of the repeat content among 4 published snake, green anole lizard genomes and the Indian cobra genome.

The average DNA base (GC) content of the *N. naja* genome was 40.46%. While MACs, representing 88% of the genome, had a GC content of 39.83%, that of MICs was 43.50% despite their containing only 12% of the genome (Welch’s two-sample *t-*test, two-sided *P* < 0.0001; Fig. 2a). Analysis of the repeat content in the Indian cobra genome in relation to other squamate reptile genomes revealed that 43.22% of the genome was repetitive (~760 Mb; Fig. 2a, Supplementary Table 4a,b, Extended Data Fig. 3 and Supplementary Note).

Whole-genome synteny comparison between the Indian cobra and prairie rattlesnake genomes revealed large syntenic blocks between the macro-, micro- and Z-chromosomes (Fig. 2b,c). We observed several fusion/fission events consistent with the difference in chromosome number between these two genomes. In particular, chromosome 4 of the Indian cobra shared syntenic regions with rattlesnake chromosomes 3 and 5, indicating a possible fusion event. In contrast, Indian cobra chromosomes 5 and 6 were syntenic to rattlesnake chromosome 5, indicating a possible fission event (Fig. 2b,c). Comparison of the Indian cobra genome to that of the more distantly related green anole lizard also showed regions of synteny and chromosomal rearrangements (Fig. 2c). Lizard chromosome 2 contained syntenic regions corresponding to chromosomes 4, 5 and 6 in the Indian cobra genome (Fig. 2c). Our synteny analysis also showed that the lizard chromosome 6 is homologous to the Z-chromosome of the Indian cobra (Fig. 2c and Supplementary Table 3b)^36^. Despite an estimated divergence time of ~280 Ma between snake and chicken (*Gallus gallus*), we observed synteny between several macro- and microchromosomes. Several regions of chicken chromosomes 1 and 2 showed syntenic blocks across Indian cobra macro-, micro- and Z-chromosomes (Fig. 2c). This indicated large-scale changes in macro- and microchromosome organization between squamate and avian genomes during evolution^31,37^.

### Gene prediction and annotation

**Fig. 3:**
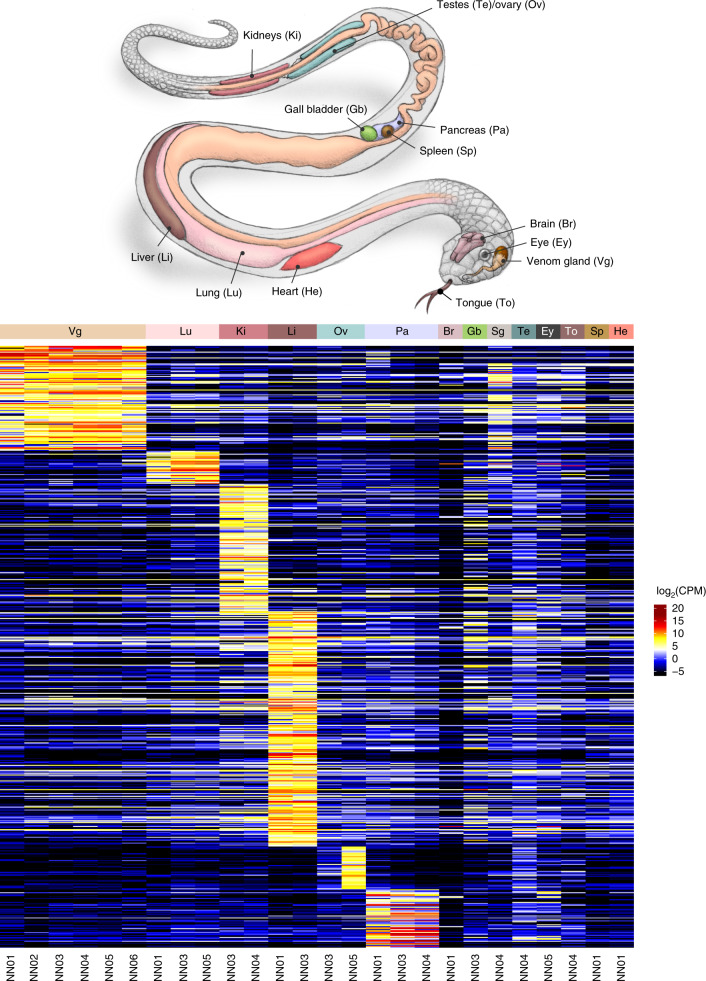
*N. naja* expression body map. Heatmap showing log_2_(CPM) values of differentially upregulated genes (DUGs); FDR < 1% across 14 tissues (sample size *n* = 6) as indicated. NN01 and NN02 correspond to *N. naja* specimens obtained from Kerala, India. NN03, NN04, NN05 and NN06 correspond to *N. naja* specimens obtained from the Kentucky Reptile Zoo. Sg, salivary gland.

We used the MAKER pipeline^38^ to annotate the genome using protein homology information, in combination with gene expression data from 14 different tissues (*n* = 26 samples; Figs. 2d and 3, Supplementary Table 1a and Supplementary Note). Overall, we predicted 23,248 protein-coding genes and 31,447 transcripts that included alternatively spliced products encoding 31,036 predicted proteins (Fig. 2d). Of the 23,248 genes, we found 22,116 (95%) on the 19 largest scaffolds corresponding to the numbered chromosomes. A total of 26,216 of the 31,036 predicted proteins (84.4%) contained a canonical start and stop codon. We identified 3,265 of 26,216 proteins (~12.5%) with an N-terminal secretion signal sequence, a feature important for venom gland toxin secretion (Supplementary Table 5). We performed extensive functional annotation of the 31,036 predicted proteins and found that 17,019 (54.8%) had a corresponding ortholog in either the Human Gene Nomenclature Committee database, NCBI’s non-redundant database or the TrEMBL (https://www.ebi.ac.uk/uniprot) database (Supplementary Tables 5 and 7b and Supplementary Note). Comparison of our annotated proteome to that of the king cobra^19^, prairie rattlesnake^35^ and green anole lizard genomes^32^ identified 26,323, 25,505 and 11,820 orthologs in those genomes, respectively.

**Fig. 4:**
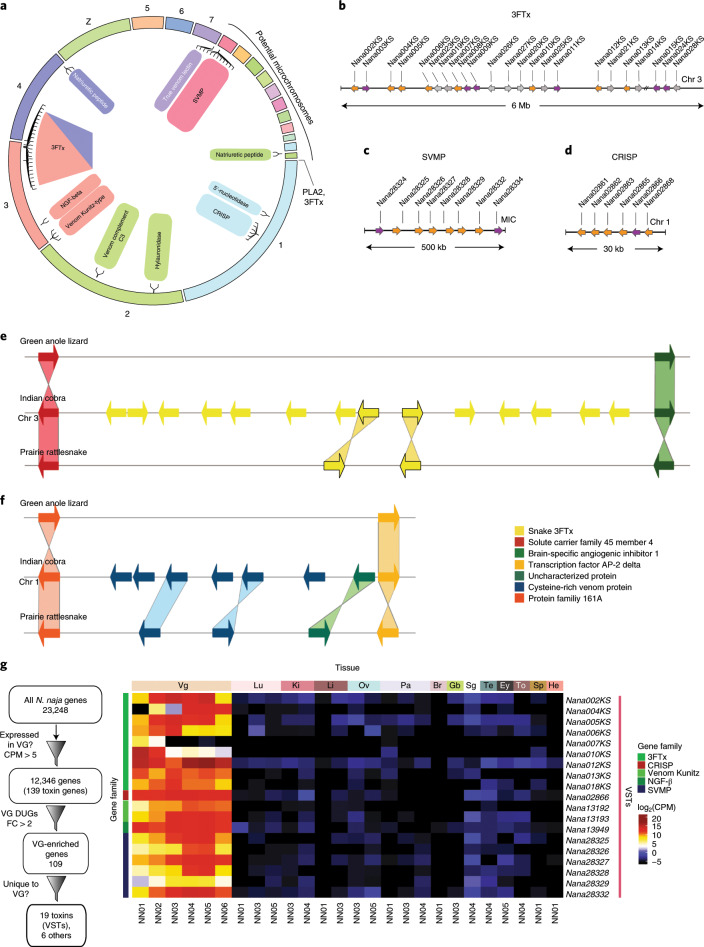
The *N. naja* venom gene repertoire. **a**, Genomic organization of *N. naja* toxin gene families. **b**–**d**, Arrayed venom gene organization of three major toxin gene families: 3FTx (**b**), SVMP (**c**) and CRISP (**d**). Genes that show venom-gland-specific expression are colored orange, and those with expression not restricted to venom glands are shown in magenta. Pseudogenes with no evidence for expression are shown in gray. **e**,**f**, Comparison showing the ancestral 3FTx (**e**) and CRISP (**f**) genes in lizard, and duplicated copies in the Indian cobra and prairie rattlesnake genomes. Orthologous gene pairs are indicated by shaded regions across the corresponding genomic regions. **g**, Schematic of filtering used to identify the 19 VSTs, and a heatmap showing the corresponding log_2_(CPM) values. NN01 and NN02 correspond to *N. naja* specimens obtained from Kerala, India. NN03, NN04, NN05 and NN06 correspond to *N. naja* specimens obtained from the Kentucky Reptile Zoo. FC, fold change; Chr, chomosome. Anatomical abbreviations as in Fig. 3.

We comprehensively annotated venom-gland-relevant genes by combining the predicted gene models with long-read sequencing data (Supplementary Table 6a and Supplementary Note), toxin gene hidden Markov models (HMM) and manual curation to identify 139 toxin genes from 33 gene families^39^ that included 19 three-finger toxins (3FTxs), 8 snake venom metalloproteinases (SVMPs) and 6 cysteine (Cys)-rich secretory venom proteins (CRISPs) (Supplementary Table 6b). Near-chromosomal assembly also allowed us to assess the genomic organization of gene families that encoded enzymatic and non-enzymatic toxin proteins involved in venom gland function. In the *N. naja* genome, 16 major toxin gene families were organized on MACs (Fig. 4a and Supplementary Table 6c). This is in contrast to the genomes of viperids *C. viridis* (prairie rattlesnake) and *Protobothrops flavoviridis* (Amami habu), where a majority of the venom gland genes were found on MICs^23,35^.

Of the 19 full-length 3FTx genes identified in the genome, 14 were clustered within a 6.3-Mb region on chromosome 3 (Fig. 4b). One 3FTx gene (*Nana001KS*) was located on chromosome 4 and the remaining four were found on an unassigned scaffold, ScVE01q_1072 (Supplementary Table 6c). Additionally, we identified 10 3FTx pseudogenes that lacked parts of the coding region and were not expressed. The second largest toxin gene family encoded by the *N. naja* genome consisted of eight SVMPs that were clustered on a MIC 1 (Fig. 4c). A cluster of six CRISPs were found on chromosome 1 (Fig. 4d). Other toxin genes, including natriuretic peptide, C-type lectin, snake venom serine proteinase (SVSP), Kunitz and venom complement-activating gene families, were found to be distributed across the 19 chromosomes while two group I Phospholipase A2 (PLA2) genes and one cobra venom factor (CVF) gene were located on an unassigned scaffold (ScVE01q_344; Supplementary Table 6c). Comparisons of venom gland genes between the *C. viridis* genome and that of the Indian cobra identified 15 toxin gene families that were unique to the Indian cobra. This included phospholipase B-like toxins and cathlecidins^40,41^ (Supplementary Table 6d). Assessment of the 139 Indian cobra venom gland toxin genes for orthologs in the king cobra showed that, while 96 genes had a match, 43 did not (Supplementary Table 6e). Although some of the 43 toxins are likely to be unique to the Indian cobra, a majority were not annotated in the king cobra genome probably due to its highly fragmented assembly (Table 2).

**Extended Data Fig. 4:**
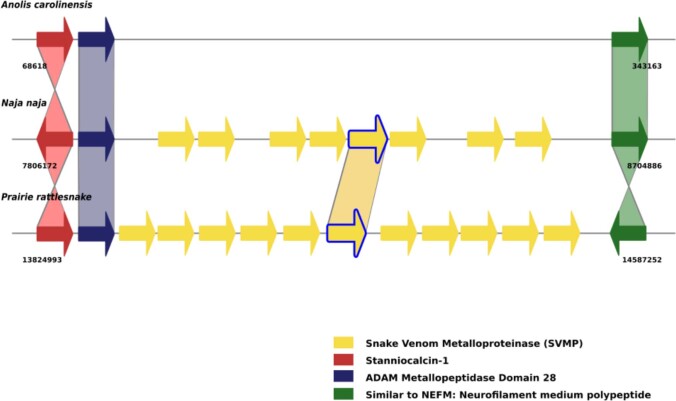
Syntenic comparisons of SVMP gene cluster. Relevant syntenic genomic regions between the Indian cobra (Nana_v5), prairie rattlesnake and green anole lizard genomes are shown. Orthologous gene pairs are indicated by shaded regions across the corresponding genomic regions. Yellow arrows with blue border indicate gene synteny, while those without colored borders represent potential species-specific duplications. SVMP, snake venom metalloproteinase.

Synteny comparisons of the major toxin gene families (3FTx, SVMP and CRISP) in genomes of the Indian cobra, prairie rattlesnake and green anole lizard revealed multiple duplication events in each family involving a paralog of non-venomous origin, leading to co-option/recruitment and expression of the duplicated gene in the venom gland (Fig. 4e,f and Extended Data Fig. 4)^19,35,42^.

### Minimal core venom-ome toxin genes

Analysis of multi-tissue transcriptome data from 26 samples representing 14 different tissues (Supplementary Table 1a) identified 19,426 expressed genes (counts per million (CPM) >1; Fig. 3 and Supplementary Table 7a,b), of which 6,601 common core genes were expressed across all tissues (Supplementary Note).

**Extended Data Fig. 5:**
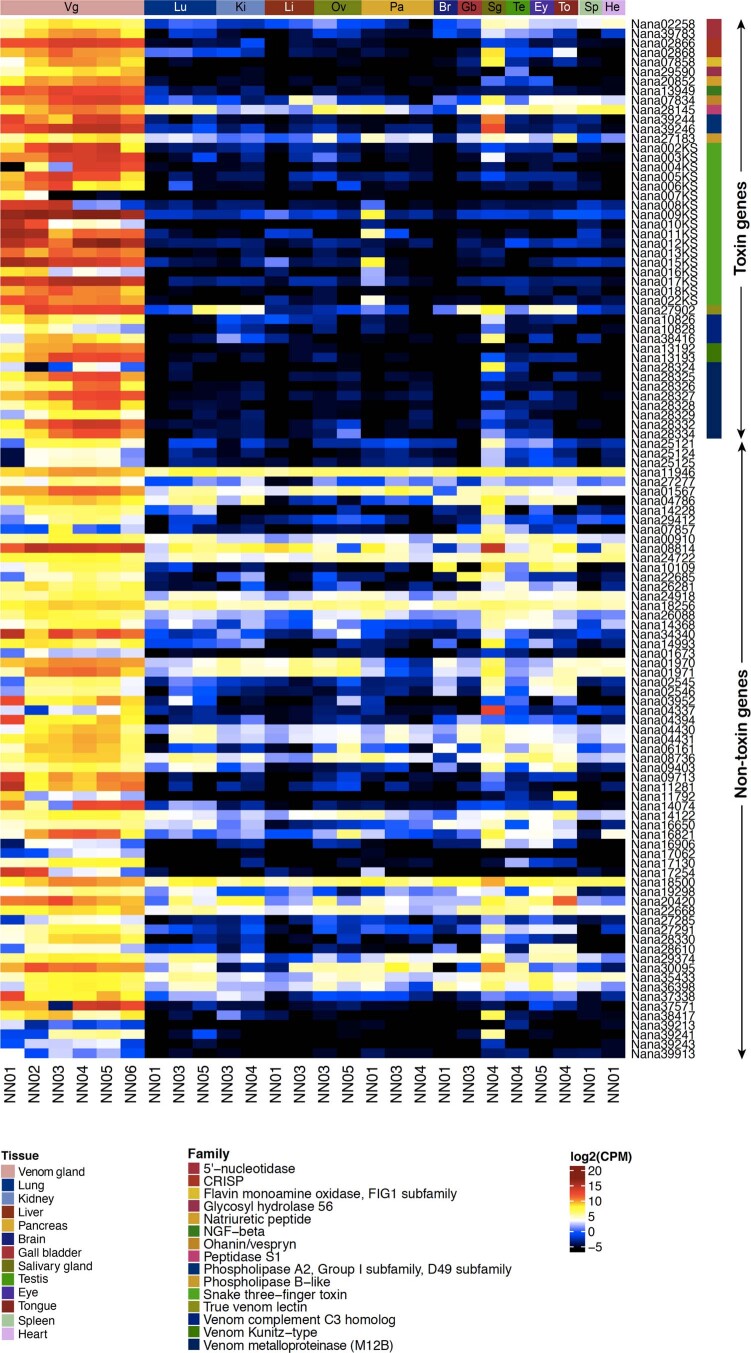
Heatmap of differentially upregulated genes in the *N. naja* venom gland transcriptome. Protein families are indicated in colored bars. NN01 and NN02 correspond to N. naja specimens obtained from Kerala, India. NN03, NN04, NN05 and NN06 correspond to *N. naja* specimens obtained from the Kentucky reptile zoo. Expression values plotted as log2 transformed CPM values with FDR cutoff set at 1% used for differential expression analysis.

The venom gland transcriptome (venom-ome) comprised 12,346 expressed genes that included 139 genes from 33 different toxin gene families. Furthermore, differential expression analysis revealed a set of 109 genes from 15 different toxin gene families that were significantly upregulated in the venom gland (fold change >2 and 1% false discovery rate (FDR); Extended Data Fig. 5 and Supplementary Table 7c), and this included 19 toxin genes that were expressed exclusively in the venom gland (Fig. 4g and Supplementary Note). These 19 VST genes are likely to encode the core venom effector toxin proteins, consisting of six neurotoxins, one cytotoxin, one cardiotoxin, one muscarinic toxin, six SVMPs, nerve growth factor (NGF-β), two venom Kunitz serine proteases and a CRISP (Supplementary Table 6b, column L). Additionally, we confirmed the presence of 16 of the 19 VSTs at the protein level using mass spectrometry (Supplementary Table 8).

### Functionally diverse 3FTxs

Three-finger toxins are short polypeptides (60–90 amino acids) that belong to a superfamily of non-enzymatic proteins found primarily in elapid snakes^43^. These small proteins are known to primarily target neuronal receptors including nicotinic acetylcholine receptors (nAChRs), muscarinic acetylcholine receptors, calcium channels and other proteins^43^. Structurally, they fold into an outstretched, three-finger-like structure where each finger contains a β-hairpin loop that extends from a disulfide bond-stabilized hydrophobic core^44,45^. 3FTxs typically contain four conserved disulfide bridges, with some containing a fifth disulfide bridge^46^. Functionally, 3FTxs are broadly classified as neurotoxins, cytotoxins, cardiotoxins and anticoagulants.

**Fig. 5:**
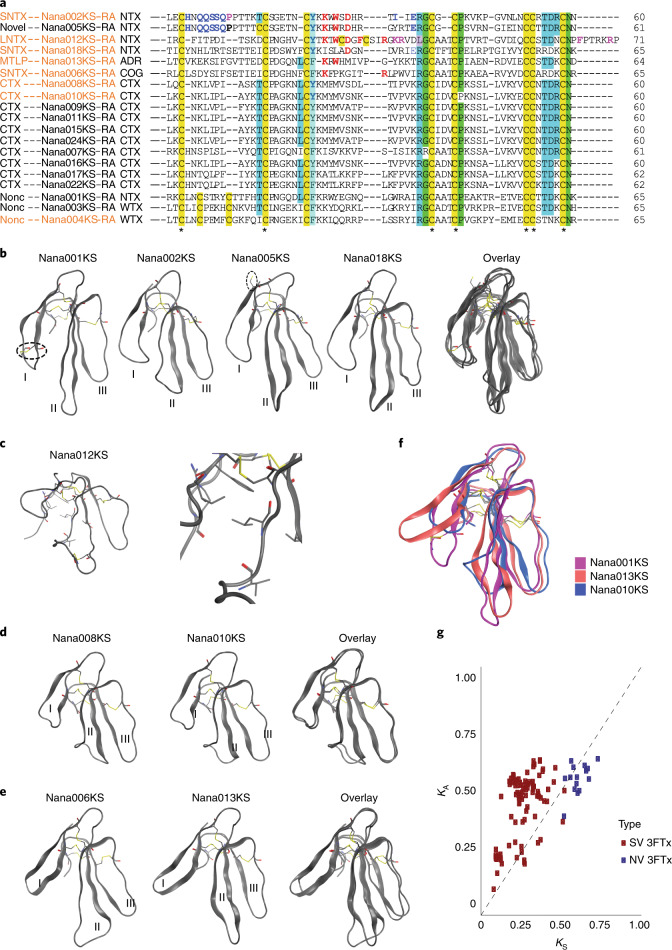
Characterization of *N. naja* 3FTx gene family. **a**, Multiple sequence alignment of the 19 3FTx proteins identified in the Indian cobra genome. Protein names in orange in the alignment indicate VSTs identified using RNA-seq. Conserved Cys residues are highlighted yellow in the alignment. **b**–**e**, Ribbon representations of representative 3FTxs from four different structural classes. Disulfide bonds are shown as sticks. The hydrophobic packing of Leu39 and surrounding residues is shown in **c**. Dashed circles highlight the additional disulfide bonds in Nana001KS, Nana012KS and the unpaired Cys in Nana005KS. **f**, Superimposition of ribbon models of Nana001KS, Nana003KS and Nana010KS highlighting the differences in loop length and conformation between the distinct classes of 3FTx found in the Indian cobra genome. **g**, Analysis of evolutionary rates on 3FTx venom genes and their non-venom paralogs. *K*_A_ and *K*_S_ values were calculated according to the Nei–Gojobori method. *K*_A_ and *K*_S_ with values <1 were not included in further analysis. SNTX, short neurotoxin; LNTX, long neurotoxin; MTLP, muscarinic toxin-like; CTX, cardiotoxin or cytotoxin; Nonc, non-conventional toxin; WTX, weak neurotoxin. SV, snake venom; NV, non-venom.

**Extended Data Fig. 6:**
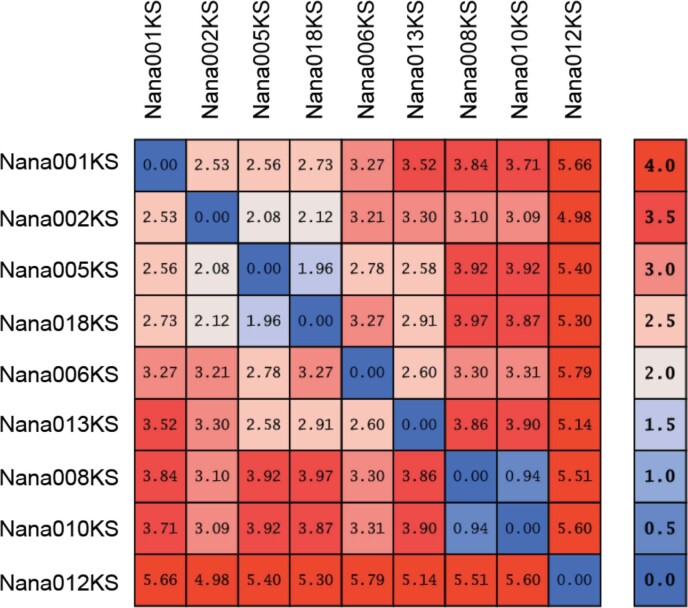
Pairwise structural comparison of representative *N. naja* 3FTxs. RMSD matrix for the structural models from 9 representative 3FTxs.

While expression of all 19 annotated *N. naja* 3FTxs was detected in the venom gland, 9 were specific to the venom gland. Of these 19 3FTxs, 14 were classified as conventional 3FTxs with 8 conserved Cys residues, while 4 3FTxs contained 10 Cys residues. Homology-based assessment enabled classification of the 3FTxs into seven neurotoxins, six cytotoxins, four cardiotoxins, one muscarinic toxin and one anticoagulant (Supplementary Table 9). The neurotoxins included two type I short-chain neurotoxins (Nana002KS and Nana005KS), known to interact with muscle nAChR, one type II long-chain neurotoxin (Nana012KS), known to target muscle and neuronal nAChRs, and three putative type III weak neurotoxins containing 10 conserved cysteines (Nana001KS, Nana003KS and Nana004KS), a characteristic feature of both non-conventional and prey-specific 3FTxs^47,48^. Nana018KS was structurally similar to haditoxin from the king cobra and known to block *α*7-nAChRs^49^. Further assessment by structural modeling classified the Indian cobra 3FTxs into four groups (Fig. 5, Extended Data Fig. 6 and Supplementary Table 10). The aromatic residue (Tyr25 or Phe27), crucial for proper folding^50^ and stability of the antiparallel β-sheet structure, was found to be conserved in all 19 3FTxs (Fig. 5a). Three of the four 10-Cys-residue-containing 3FTxs were non-conventional 3FTxs containing a pair of additional cysteines in loop I, resulting in a longer N terminus loop that could potentially also play a role in stabilizing this loop and contribute to toxin function^47^ (Fig. 5a,b). The other 10-Cys 3FTx was a long-chain neurotoxin that contained a pair of additional cysteines in loop II (Nana012KS). The charged amino acid residue Arg39, which typically stabilizes native toxin conformation by forming a salt bridge with the C terminus^51^, was conserved in all identified 3FTxs except in Nana012KS, where it was replaced by a leucine residue (Fig. 5a). The homology model of this protein indicated that Leu39 may exhibit van der Waals interactions and form part of a hydrophobic core comprising Ile35, Phe4, Thr22 and Arg68 (Fig. 5c). Two of the cytotoxins, Nana008KS and Nana010KS, had shorter loops I and III compared to the other 3FTxs (Fig. 5d).

Of note, Nana005KS contained nine Cys residues and such unusual 3FTxs have been found in only two other elapids, *Micrurus lemniscatus* and *Micrurus altirostris*^52^. This free Cys residue at position 16 that precedes the conserved second Cys in loop I (Fig. 5b) probably facilitates the formation of covalent homo- or heterodimeric 3FTxs (Supplementary Table 10). Nana005KS was closely related to the short-chain 3FTx Nana002KS and contained a majority of the residues required for activity against muscle nAChRs. For instance, the presence of the positively charged residues Lys25, Lys26 and Arg32 indicates that these toxins might be crucial for envenomation in mammals. In particular, the guanidyl group of Arg32 mimics acetylcholine forming a cation–π interaction with α–α and α–δ interfaces in the nAChRs^53^.

**Extended Data Fig. 7:**
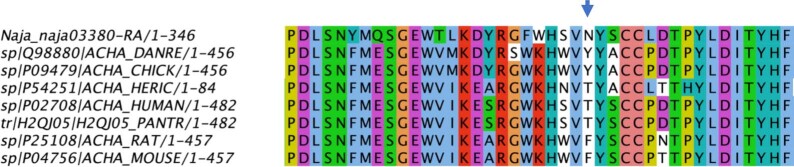
nAChR polymorphism in Indian cobra. Multiple sequence alignment showing the region surrounding the alpha neurotoxin binding site in nAChR of seven vertebrate animals and *N. naja* nAChR (Nana03380-RA) identified a SNP at residue 189 in the *N. naja* nAChR indicated by the blue arrow. nAChR – nicotinic acetylcholine receptor; DANRE *– Danio rerio*; CHICK, *Gallus gallus*; HERIC, *Herpestes ichneumon*; HUMAN, *Homo sapiens*; PANTR, *Pan troglodytes*; RAT, *Rattus norvegicus*; MOUSE, *Mus musculus*.

Acquired mutations affecting the neurotoxin binding site of nAChR have rendered certain species, such as the Egyptian mongoose (*Herpestes ichneumon*), immune to snake venom^54–56^. Comparison of *N. naja* nAChR sequence and *H. ichneumon* nAChR and other representative mammalian species showed that the *N. naja* nAChR carries a key p.Phe189Asn alteration in the α-neurotoxin site, known to result in decreased sensitivity to short- and long-chain neurotoxins^55,56^ (Extended Data Fig. 7).

In the final group of 3FTxs, Nana013KS was structurally similar to AncTx-1 (ref. ^57^), a synthetic ancestral toxin known to interact with β-adrenergic G-protein-coupled receptors. The 3FTx encoded by *Nana006KS* was found to be a close homolog of ringhalexin, an inhibitor of the extrinsic tenase coagulation complex, from *Hemachatus haemachatus*, the African ringhals cobra^58^ (Fig. 5e and Supplementary Table 9). Additionally, we found Nana017KS to be 97% similar to a recently reported *Naja atra* (*μ*-EPTX-Na1a)^59^ Nav1.8 voltage-gated sodium channel inhibitor.

To understand the consequences of genetic variation in the 3FTx family, we assessed the rate of evolution of the major toxin families by computing the numbers of synonymous (*K*_S_) and non-synonymous (K_A_) nucleotide substitutions per site for each pairing of toxin gene and its non-toxin paralog. The *K*_A_/*K*_S_ substitution ratio for the 3FTx toxin genes was 2.034 (±0.818), while that of the non-toxin paralogs (Ly-6/UPAR domain-containing genes) was 0.894 (±0.103; Fig. 5g). The observed high *K*_A_/*K*_S_ ratio (>1) indicated diversifying selection leading to rapid divergence and functional diversification of the venom-gland-specific 3FTx genes.

### Indian cobra SVMP, CRISPs, PLA2, CVF and growth factor genes

**Extended Data Fig. 8:**
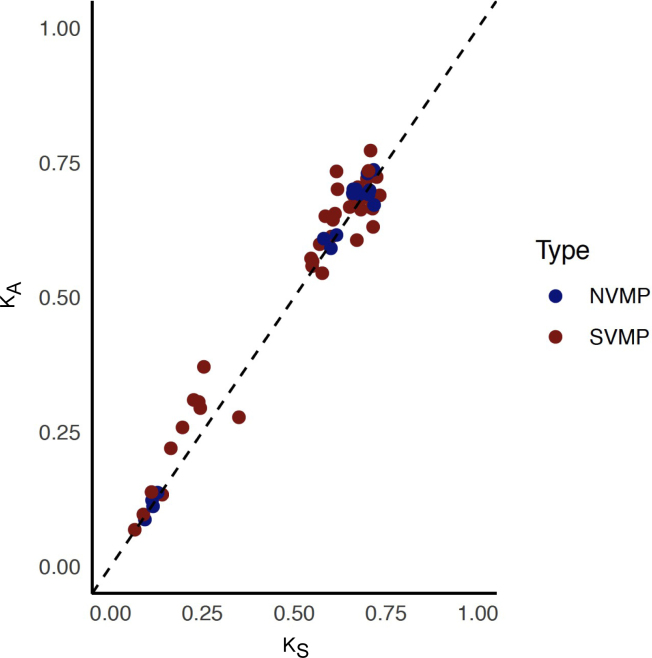
Molecular evolution of Indian cobra SVMP genes. K_A_ and K_S_ values were calculated according to the Nei-Gojobori method. K_A_ and K_S_ with values < 0.1 were not included in further analysis for reliable analysis. NVMP, Non-venomous metalloproteinase genes; SVMP, Snake venom metalloproteinase genes.

**Extended Data Fig. 9:**
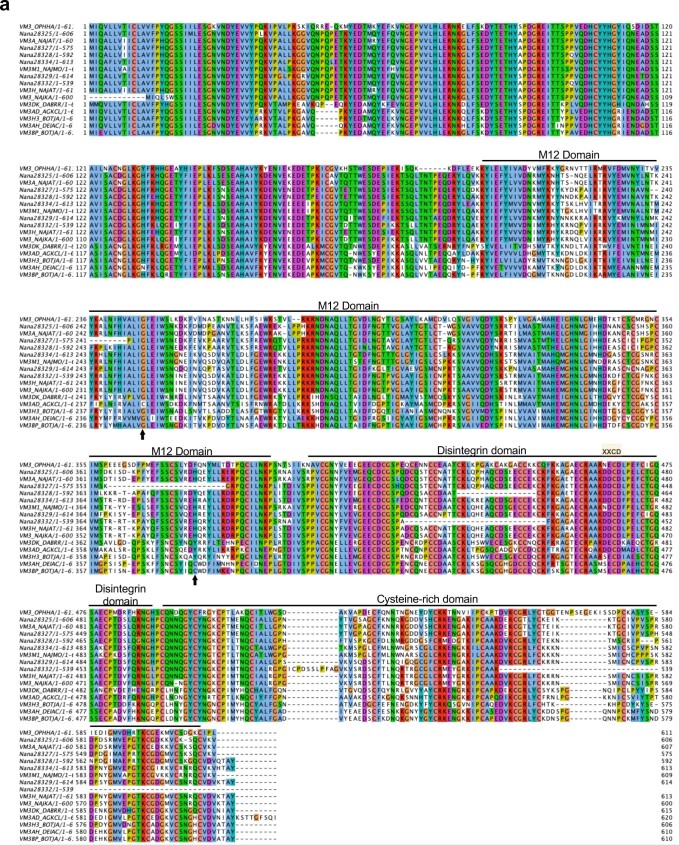
SVMP expression and comparative protein sequence alignment. **a**, Multiple sequence alignment of SVMP proteins from the Indian cobra, and representative SVMPs from other Elapid and Viperid species and human ADAM28. Arrows indicate additional cysteine residues typically present in the M12 domain of Viperidae SVMPs. **b**, Phylogenetic tree reveals distinct clusters formed by elapid and viperid SVMPs. The bar indicates 0.03 substitutions per nucleotide position. Elapid species - OPHHA, *Ophiophagus hannah;* NAJAT, *Naja atra*; NAJMO, *Naja mossambica*; NAJKA, *Naja kaouthia*; Viperid species - DABRR, *Daboia ruselli*; AGKCL, *Agkistrodon contortrix laticinctus*; DEIAC, *Deinagkistrodon acutus*; BOTJA, *Bothrops jararaca;* HUMAN, *Homo sapiens*.

We detected six venom-gland-specific SVMPs that belonged to the P-III class of metalloproteinases (with metalloproteinase/disintegrin/Cys-rich domains) known to be involved in the induction of hemorrhage, inflammation, apoptosis, prothrombin activation and inhibition of platelet aggregation^60^. The Indian cobra SVMPs were found to evolve less rapidly than 3FTx genes, because the *K*_A_/*K*_S_ ratio for the venom-gland-specific SVMPs was 1.070 (±0.137) when compared to 0.998 (±0.049) observed for metalloprotease domain-containing paralogs (Extended Data Fig. 8). The Indian cobra SVMPs formed a separate cluster compared with the viperid SVMPs (Extended Data Fig. 9). In agreement with this, a seventh Cys residue in domain M12, involved in the disulfide bond exchange for autolysis during secretion or formation of the biologically active disintegrin/Cys-rich domain typical of Viperidae SVMP-PIIIs^60^, was absent (Extended Data Fig. 9).

We also detected six CRISPs in the venom gland transcriptome that were highly conserved across different snake species (Supplementary Fig. 1). Venom CRISPs have a wide variety of biological effects, including blockade of K^+^ and/or Ca^2+^ currents in neurons and blockage of vascular smooth muscle contraction^61^. Two of the five CRISPs were homologous to venom CRISPs from the Chinese cobra, *Naja atra* (natrin), and the monocled cobra, *Naja kaouthia* (UniProtKB: Q7T1K6). Expression of the *N. naja* natrin homolog (*Nana02866*) was venom gland specific, and it probably functions as a Kv1.3 potassium channel blocker^62^.

Additionally, two group I secretory acidic PLA2 genes (*Nana39244* and *Nana39246*) that were highly expressed in the venom and salivary glands showed a high degree of similarity to other elapid venom PLA2s, and contained the characteristic calcium-binding (XCGXGG) and catalytic (DXCCXXHD) motifs^63^ (Supplementary Fig. 2 and Supplementary Note).

In addition to the major elapid toxin families described above, we detected transcripts from other toxin gene families including hyaluronidases, phospholipase B-like genes, cathelicidins, ohanin (known to induce hypolocomotion and hyperalgaesia)^64^ and 5′ nucleotidases (Supplementary Note). We also detected l-amino acid oxidase (LAAO) (*Nana07858*), which is involved in platelet aggregation, edema and hemorrhage. Furthermore, we identified two full-length c-type natriuretic peptide genes, *Nana20849* and *Nana20852*, in the venom-ome. Of the three Kunitz serine protease inhibitors detected in the venom-ome, two were VST gene products (Nana13192 and Nana13193) and these probably function to inhibit serine proteases acting in the hemostatic system^65^. In addition, two full-length cystatin genes, *Nana15538* and *Nana35841*, were also found expressed in the venom-ome.

Cobra venom factor is a non-lethal protein that resembles the complement C3 protein in structure and function^66^. Previously, the complete structure of one CVF gene from *N. Naja* has been reported^67^. In the present study, we identified three CVF genes (*Nana10828*, *Nana38416 and Nana10826*) in the *N. naja* genome. *Nana38416 and Nana10828* contained 40 exons and spanned ~118 and ~75 kb, respectively, on chromosome 2. Isoform sequencing (Iso-seq) data confirmed the expression of the full-length transcripts corresponding to *Nana10828* and *Nana38416*. Though the 5′ genomic structure of *Nana10826* was not fully resolved in the currently assembly, the expression of the full-length transcript of *Nana10826* was confirmed by iso-seq. Protein sequence alignment showed that Nana38416 was 96% similar to CVF (UniProtKB: Q91132) from *N. kaouthia*, while Nana10828 was 99% similar to a previously characterized *N. naja* C3 complement component protein^68^.

In addition to toxin components, we also identified four full-length *PDGF*/*VEGF*-growth factor genes including vascular endothelial growth factor-1 (*VEGF1*; *Nana01393*), *VEGFC* (*Nana18254*), platelet-derived growth factor (*PDGF*; *Nana34300*), placenta growth factor (*PGF*; *Nana05337*) and an insulin growth factor (*IGF*) gene and *Nana04360* in the venom-ome. Furthermore, we also identified a venom-gland-specific nerve growth factor (*NGF-β*; *Nana13949*) that exhibited high homology to NGFV2 from the spitting cobra *Naja sputratrix*.

## Discussion

Much of our current understanding of snake venom is based on proteomic studies that have provided only a partial picture of its components^69–71^. A comprehensive catalog of venom proteins, their expression and coding sequence is fundamental to developing a safe and effective antivenom^15,72^. Also, such a detailed catalog of venom components will be valuable for drug candidate prospecting.

Using next-generation sequencing in combination with emerging genomic technologies, we have generated a de novo high-quality *N. naja* reference genome^73^. The near-chromosomal assembly revealed regions of synteny between reptilian and avian genomes, consistent with their evolutionary trajectories. The high contiguity of our genome enabled visualization of the striking differences in venom gene organization between elapid and viperid snake genomes. The locations of the major toxin gene families in the *N. naja* genome on MACs was in contrast to their presence on MICs in *P. flavoviridis* and *C. viridis*^23,35^ genomes, indicative of the differences in their chromosome and venom evolution.

**Fig. 6:**
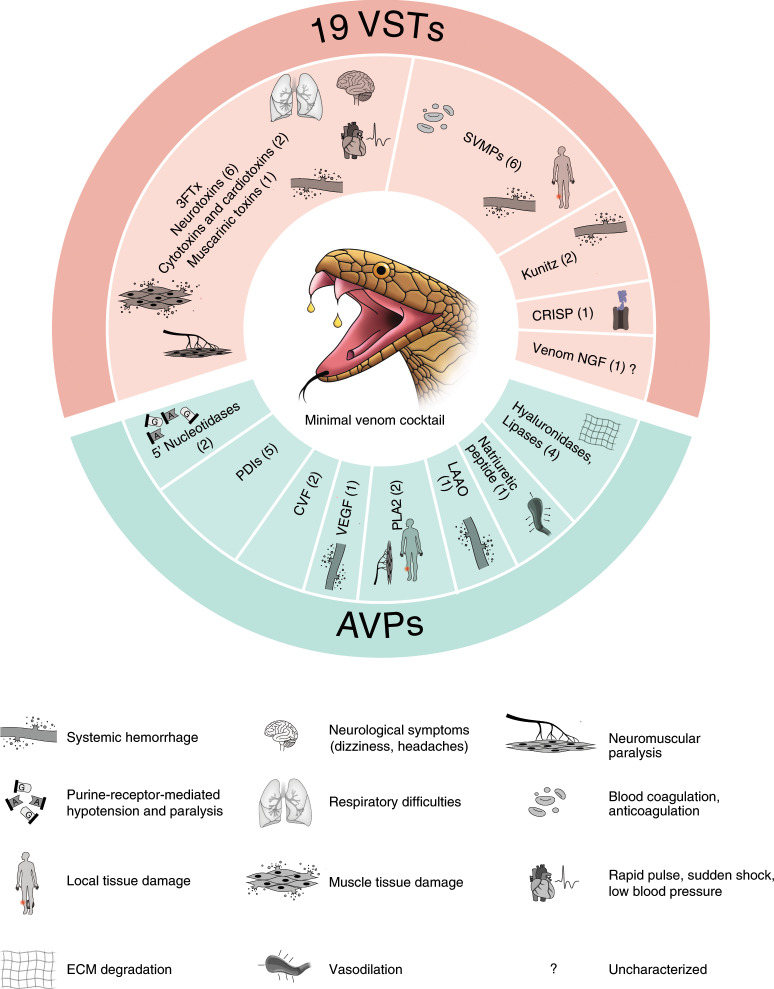
*N. naja* minimal venom cocktail. The 19 VSTs, accessory venom proteins (AVPs) and their primary physiological targets. ECM, extracellular matrix; PDIs, protein disulfide isomerases. See Supplementary Table 6b (column L) for VST and AVP gene names.

**Extended Data Fig. 10:**
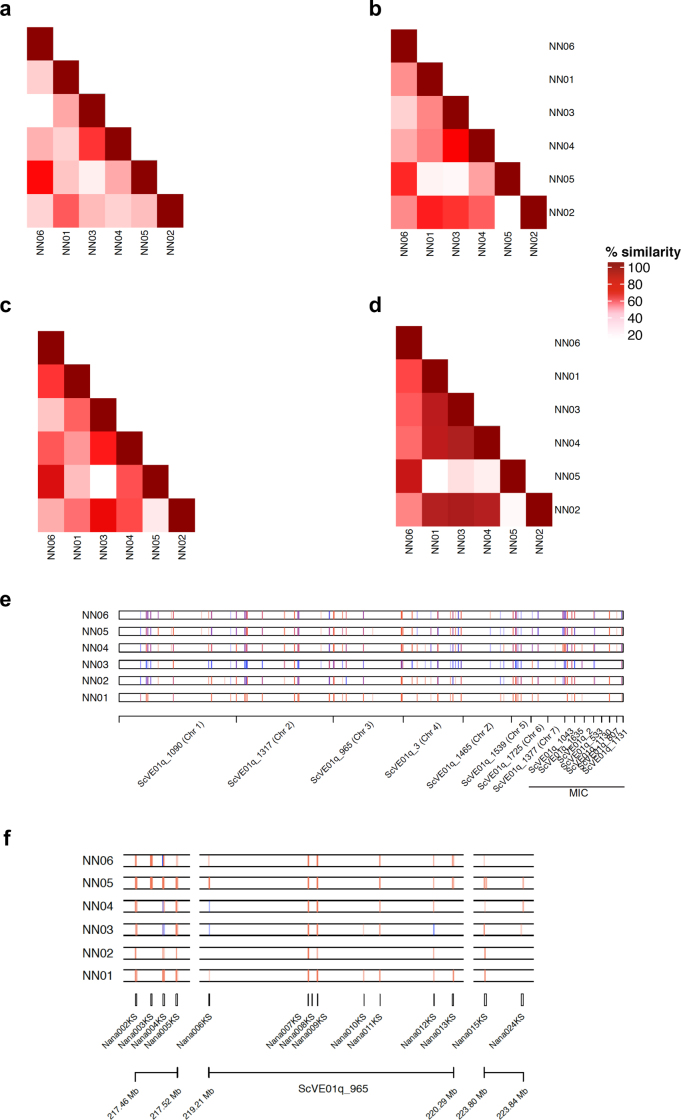
Genetic polymorphisms in 6 *N. naja* specimens. **a**–**d**, Pairwise similarity (PWS) matrices based on (**a**), all genome-wide protein-altering variants, (**b**), all venom gland-expressed genes, (**c**), core venom-ome genes, and (**d**), all 3FTx genes identified in this study. **e**, Distribution of protein altering variants in 106 venom gland-specific toxin genes. **f**, Distribution of protein altering variants in 3FTx genes located on chromosome 3 across all six study animals (NN01-NN06). Within each track in (b-f), homozygous variants are shown as blue vertical lines while heterozygous variants are shown as red vertical lines. NN01 and NN02 correspond to *N. naja* specimens obtained from Kerala, India. NN03, NN04, NN05 and NN06 correspond to *N. naja* specimens obtained from the Kentucky reptile zoo. MIC, microchromosomes.

Overall, we found evidence for expression of 12,346 genes that constitute the venom-ome, and this included 139 toxin genes of which 19 were designated as VSTs based on their venom-gland-specific expression. Additionally, well-known modulators of venom function such as CVF, coagulation factors, protein disulfide isomerases, natriuretic peptides, hyaluronidases, PLA2s, phospholipase B-like genes, LAAO, vascular endothelial growth factor (VEGF) and 5′ nucleotidases were also found to be highly expressed in the venom gland. It is likely that these genes, together with the 19 VSTs, form the core toxic effector components of the venom and induce a wide range of symptoms including cardiovascular dysfunction, muscular paralysis, nausea, blurred vision and systemic effects such as hemorrhage^6^ (Fig. 6). We propose that neutralization of these core venom effectors using antibodies would be an effective therapeutic strategy. Furthermore, given the variation in venom composition, cataloging the venom gland gene repertoire and its variation (Extended Data Fig. 10 and Supplementary Note), both within and across different snake species, will be important for developing a broadly efficacious antivenom^74,75^.

Snake antivenom is made by immunizing large mammals, such as horses, with extracted snake venom as antigen^12^. Such horse-derived antibodies show variation in efficacy due to expected differences in the horse antibody response following antigen challenge^6,13^. Moreover, the heterologous nature of the antibodies leads to adverse treatment-related side effects^6,13^. Starting with a complete catalog of VSTs, synthetic venom of a defined composition can be generated using recombinant protein expression technologies^76^. Such a cocktail of recombinant core venom VST proteins can be used to raise specific antibodies in horses, tested for neutralizing activity, rapidly cloned/synthesized and humanized to produce the next generation of antivenoms^14,16,77^. Alternatively, recombinant venom proteins can be used as baits against antibody phage libraries to obtain toxin-neutralizing, activity-tested humanized synthetic antibodies^16,72,78^.

Genome-guided recombinantly produced venom proteins can be also used to characterize and improve existing horse-derived antivenom. In this paradigm, antibodies can be raised against recombinant venom toxins that are non-immunogenic in horses and used to supplement current antivenom cocktails to improve their efficacy. Alternatively, single-cell sequencing of B cell repertoires from horses immunized with extracted or synthetic venom can be performed to identify putative neutralizing antibodies^79^. This information can be used to rapidly synthesize, humanize and identify toxin-neutralizing antibodies for the generation of synthetic antivenom.

As more high-quality snake genomes are completed and venom gland VSTs are cataloged, synthetic antibodies directed against key species-specific toxins identified from such genome initiatives can be combined to create potent broad-spectrum antivenom^80^. Furthermore, antibodies targeting different epitopes on recombinant toxins can be developed using phage display. These recombinant toxin-directed antibodies can be combined to generate an antivenom that is likely to be more efficacious^81–84^.

Primary cultures of venom gland cells^85^ or the recently developed venom gland organoid cultures (Post, Y. et al., personal communication), in combination with genomic information, can provide an alternative, viable source of venom antigens for antivenom development (Supplementary Note).

We believe the Indian cobra reference genome and the analysis presented here will facilitate innovations in antivenom development. The genome and the associated predicted proteome will serve as a powerful platform for evolutionary studies of venomous organisms. More importantly, the comprehensive catalog of the venom proteins presented here should enable drug development, in particular to treat hypertension, pain and other disorders^86–88^.

## Methods

### Karyotyping

Briefly, blood from a female animal (NN03) was collected in a sodium-heparin vacutainer (Becton–Dickinson) and used for short-term (72-h) lymphocyte cultures as previously described^89^. We used phytohemagglutinin (PHA from *Phaseolus vulgaris*, 20 μg ml^–1^, Sigma Aldrich) as the mitogen. Additionally, we established primary fibroblast cultures under sterile conditions using small pieces (0.5 mm^2^) of ovarian tissue from NN03. The fibroblasts were incubated in MEM alpha containing nucleosides and GlutaMax (Thermo Fisher), supplemented with 20% fetal bovine serum (Atlanta Biologicals) and antibiotic-antimycotic (Thermo Fisher) at 30 °C with 5% CO_2_. Metaphase chromosomes were obtained from both lymphocyte and fibroblast cultures by arresting cells with demecolcine (KaryoMax, Thermo Fisher; final concentration, 0.1 μg ml^–1^), followed by hypotonic treatment with Optimal Hypotonic Solution (Rainbow Scientific) and fixation in methanol/acetic acid (3/1). Metaphase spreads were prepared on precleaned wet glass slides at room temperature. Chromosomes were stained with 5% Giemsa (karyoMax, Thermo Fisher) in GURR buffer (Gibco). At least 30 metaphase spreads were captured and analyzed for karyotyping using an Axioplan2 microscope (Zeiss) and Ikaros (MetaSystems) software.

### Flow cytometry-based genome size estimation

We estimated genome size by flow cytometry using a previously described protocol^90^. Whole blood cells of the Indian cobra (NN03) were cultured for 4 d in a medium consisting of MEM alpha with nucleosides and GlutaMax (Thermo Fisher), 30% fetal bovine serum (Atlanta Biologicals) and 1% PHA (1 mg ml^–1^, Sigma Aldrich) at 30 °C. Blood cells were washed six times in sterile water and then fixed in cold ethanol. Horse (*Equus caballus*) lymphocytes from a female were separated from erythrocytes with a Lymphoprep (Stemcell Technologies) before being cleaned and fixed in the same manner as the Indian cobra cells. Next, both cell types were diluted to a concentration of 1 × 10^6^ cells ml^–1^ and stained with propidium iodide (PI, Thermo Fisher). After staining, the Indian cobra and horse cells were mixed 1/1 and analyzed on a Becton–Dickinson Accuri C6 personal flow cytometer. Two peaks were observed based on the amount of PI absorbed by the cells of either species, and genome size was estimated using the formula: genome size (cobra) = PI (cobra)/PI (horse) × genome size (horse), where PI (cobra) denotes the median amount of PI absorbed by cobra cells, PI (horse) denotes the median amount of PI absorbed by horse cells, and genome size (horse) is the expected size of the horse genome of 2.5 Gb (EquCab3, GCA_002863925.1)^91^.

### Samples and nucleic acid preps

A total of six animals—two from Kerala, India (NN01 and NN02) and four unrelated animals from the Kentucky Reptile Zoo, KY (NN03, NN04, NN05 and NN06)—were part of the study (Supplementary Table 1a). Two animals from India were fresh road kills that were submitted for post-mortem analysis to the Wayanad wildlife sanctuary forest veterinary officer, and were permitted to be used for this study under order no. WL10–12401/2017 from the Kerala forest and wildlife chief warden. A summary of tissues collected and processed from each animal is shown in Supplementary Table 1a. Genomic DNA was prepared using the Qiagen Magattract HMW DNA kit (Qiagen). RNA was extracted using the Qiagen RNeasy kit (Qiagen).

### Genome sequencing

Libraries for ONT, PacBio SMRT and Illumina sequencing were constructed as per the manufacturers’ instructions using high-molecular weight (HMW) DNA extracted from animal NN01 liver and kidney. A total of 71.23-Gb (~40×) PacBio RSII/Sequel, 61.8 -Gb (~34×) ONT and 117.5-Gb (~64×) Illumina (2 × 150-base pairs (bp)) data were generated. Additionally, paired-end Illumina sequencing data (2 × 150 bp) were generated for each study animal as indicated in Supplementary Table 1b.

### BNG data generation

Purified DNA extracted from muscle tissue from animal NN01 was embedded in a thin agarose layer, labeled and counterstained following the nick, label, repair and stain (NLRS) or direct label and stain (DLS) Reagent Kit protocol (BNG). Samples were then loaded onto Saphyr chips and run on the Saphyr imaging instrument (BNG). A total of 814 and 580 Gb of optical map data were generated using the NLRS or DLS protocol, respectively. De novo genome assembly using the BNG Access software for the NLRS or DLS optical map data produced assemblies consisting of 3,921 and 1,477 scaffolds with scaffold N50 of 0.88 and 16.33 Mb, respectively. Additionally, we performed BNG DNA labeling using DNA extracted from the ovary of NN05 for use in scaffolding with the 10x Genomics–derived de novo assembly. A total of 400 Gb of BNG data were generated for this animal using the DLS protocol, resulting in a de novo optical map assembly consisting of 1,338 scaffolds and a scaffold N50 of 15.92 Mb.

### Chicago library preparation and sequencing

Three Chicago libraries were prepared as described previously^28^ using DNA from muscle tissue corresponding to animal NN01 (Dovetail Genomics). Briefly, for each library, ~500 ng of HMW gDNA (mean fragment length, 75 kb) was reconstituted into chromatin in vitro and fixed with formaldehyde. Fixed chromatin was digested with DpnII, the 5′ overhangs filled in with biotinylated nucleotides and free blunt ends were then ligated. After ligation, cross-links were reversed and the DNA purified from protein. Purified DNA was treated to remove biotin that was not internal to ligated fragments. The DNA was then sheared to ~350 bp mean fragment size and sequencing libraries were generated using NEBNext Ultra enzymes and Illumina-compatible adapters. Biotin-containing fragments were isolated using streptavidin beads before PCR enrichment of each library. The libraries were sequenced on an Illumina platform. We produced 2 × 150-bp reads and about 525 million, 489 million and 516 million reads for libraries 1, 2 and 3, respectively. Together, these Chicago library reads provided ~250× (459-Gb) physical coverage of the genome (1–50 kb pairs).

### Hi-C library preparation and sequencing

Two Dovetail Genomics Hi-C libraries were prepared as described previously^29^ using muscle tissue from animal NN01. Briefly, for each library, chromatin was fixed in place with formaldehyde in the nucleus and then extracted. Fixed chromatin was digested with DpnII, the 5′ overhangs filled in with biotinylated nucleotides and then free blunt ends were ligated. After ligation, cross-links were reversed and the DNA purified to remove proteins. Purified DNA was treated to remove biotin that was not internal to ligated fragments. The DNA was then sheared to ~350-bp mean fragment size and sequencing libraries were generated using NEBNext Ultra enzymes and Illumina-compatible adapters. Biotin-containing fragments were isolated using streptavidin beads before PCR enrichment of each library. The libraries were sequenced on an Illumina platform to generate 242 million and 291 million 2 × 150-bp reads for libraries 1 and 2, respectively. Together, these Dovetail Hi-C library reads provided ~89×  (159 Gb) physical coverage of the genome (1–50 kb pairs).

### 10x Genomics library generation and sequencing

High-molecular-weight DNA was extracted from a female (NN05) ovary using the 10x Chromium HMW DNA extraction protocol (10x Genomics). With 1 ng of template, DNA whole-genome sequencing libraries were prepared using the Chromium Genome Library and Gel Bead Kit v.2 (10x Genomics, no. 120258), Chromium Genome Chip Kit v.2 (10x Genomics, no. 120257), Chromium i7 Multiplex Kit (10x Genomics, no. 120262) and Chromium controller (10x Genomics). In total, 761.34 million linked-reads (114 Gb, 2 × 150-bp) were generated providing a raw coverage of ~65×.

### Scaffold to chromosome mapping

We used the synteny between *Elaphe* chromosomes and the *N. naja* Nana_v5 genome to assign scaffolds to chromosomes. Briefly, 105 cDNA sequences corresponding to *E. quadrivirgata* chromosomes^31^ were downloaded from the NCBI nucleotide database. The cDNA sequences were aligned to the *N. naja* Nana_v5 genome assembly using Exonerate^92^. The chromosome number corresponding to the *Elaphe* cDNA marker was assigned to the best matching Nana_v5 scaffold based on the highest sequence alignment score.

### Snake chromosome laser capture microdissection and SChrom-seq

Chromosome slides for laser microdissection were prepared on Leica 0.9-µm POL-membrane frame steel frame slides (Leica, no. 11505188) following the manufacturer’s recommended protocol. About 10 µl of fixed and previously tested metaphase cell suspensions from ovarian (NN03) fibroblasts was dropped on a dry POL-membrane slide, allowed to spread by gravity and dried overnight. Air-dried slides were placed in 50 ml of Giemsa solution (1 ml of 5% Giemsa staining solution per 50 ml of GURR buffer (Gibco)) for 10 min, washed three times in sterile water and air-dried. Laser confocal microscopy-enabled dissection of individual chromosomes was done with the Leica LMD 6 Laser Microdissection microscope (150× dry-immersion objective; HC PL FLUOTAR ×150/0.9 numerical aperture) and software. A total of 104 dissections were performed. Each sample tube containing microdissected chromosomes was lysed to release genomic DNA, and whole-genome amplification performed to amplify the DNA and create libraries with the SMARTer PicoPLEX DNA-seq kit (Takara Bio). The resulting 250-bp single-end libraries were further amplified exponentially with primers containing unique Illumina dual-index barcodes suitable for Illumina sequencing. Data from each library were mapped to a combined reference genome including GRCh38 and snake (Nana_v5) using Burrows–Wheeler aligner (BWA)^93^ with default options. Mapped reads were sorted and duplicates marked with PicardTools (http://broadinstitute.github.io/picard/). Reads mapping to multiple loci, GRCh38, a combined bacterial sequence database or those with a mapping quality below 10 were discarded. All remaining mapped reads were then used to calculate total counts per chromosome. Coverage data were generated with GATK^94^ and binned using 100-kb windows.

### Synteny mapping and dot-plot analysis

The repeat-masked *C. viridis* (prairie rattlesnake) and *A. carolinensis* genomes were aligned to Nana_v5 chromosomes with CoGe’s SynMap program using LAST^95^. The 10x-BNG hybrid scaffolds and Nana_v5 scaffolds (>1 Mb) were aligned with Symap (v.4.2) using default parameters^96^. Chromosome painting of *N. naja* chromosomes with prairie rattlesnake chromosomes was performed using the SatsumaSynteny2 script with default parameters^97^.

### RNA-sequencing

Ribonucleic acid was extracted from the venom gland, accessory gland, heart, lung, spleen, brain, ovary, testes, gall bladder, pancreas, kidney, liver, eye and tongue (Supplementary Table 1a) using the Qiagen RNeasy Kit (Qiagen). PolyA RNA-sequencing (RNA-seq) libraries were prepared with 1 µg RNA from each tissue using the Illumina TruSeq stranded messenger RNA kit and sequenced on HiSeq (Illumina).

### Iso-seq analysis

Total RNA from venom gland was used to generate cDNA and PacBio iso-seq libraries as per the manufacturer’s instructions. Size selection of libraries was performed using the BluePippin system (Sage Science). Sequencing was performed on a PacBio RSII/Sequel (Supplementary Table 5a). Full-length consensus isoform sequences were generated using PacBio’s SMRT portal and SMRTLink for the RSII and Sequel data, respectively. Arrow (https://github.com/PacificBiosciences/GenomicConsensus) was used to polish the consensus isoforms. In total, 101,761 transcript isoforms were processed by the pipeline. Full-length transcripts (that contained a complete open reading frame) were then aligned to the genome using GMAP with parameters specific to long-read alignment^98^. Complete open reading frames were directly annotated with tBLASTx (v.2.2.29+)^99^ against NCBI NR and TrEMBL using the same pipeline as done with whole-genome annotation. We used these data to manually verify and correct venom-gland-specific toxin gene annotations (Supplementary Note).

### Repeat element identification

We identified the repetitive elements in the genome by combining both homology-based and de novo predictions. Next, we used a previously described reptile-specific repeat library^20^ with RepeatMasker (v.4.0.7) (http://www.repeatmasker.org) to annotate repetitive elements in the Indian cobra genome. We then used RepeatModeler (v.1.0.11) (http://www.repeatmasker.org/RepeatModeler.html) to construct the species-specific repeat sequence libraries for the Indian cobra, and then used these as a query to identify repetitive elements using RepeatMasker. Finally, we retrieved a non-redundant annotation for each species after combining all the annotation results using the reptile-specific libraries and de novo repeat library.

### Venom gene synteny analysis

BLASTn was used to identify homologs of all *N. naja* toxin genes (Supplementary Table 6b) in the *Anolis* and *C. viridis* genomes. BLASTn hits were then filtered using the following parameters: query coverage ≥70% and identity ≥80%. Synteny was then plotted using a publicly available Python script for gene synteny visualization (https://github.com/biopython/biopython/blob/master/Doc/examples/Proux_et_al_2002_Figure_6.py).

### Differential gene expression analysis

After adapter trimming and quality filtering, reads were aligned to Nana_v5 using STAR (v.2.6.0a)^100^ with default parameters. Gene counts per gene across all samples were calculated using featureCounts^101^. Raw gene counts were then normalized between samples using the CPM normalization method from the EdgeR package^102^ (Supplementary Table 7b–h). Differential expression analysis was then performed using EdgeR (fold change >2 and 1% FDR), and differentially upregulated genes (DUGs) were filtered for further analysis for each tissue that had a replicate. Gene Ontology-term enrichment analysis of DUGs was performed using EnrichR^103,104^ (Supplementary Note).

### Liquid chromatography–tandem mass spectrometry analysis of venom

Pooled Indian cobra venom samples obtained from Kentucky Reptile Zoo (10 μg per lane) were reduced with DTT (10 mM, 60 min at 37 °C), alkylated with iodoacetamide (20 mM, 15 min at room temperature) and separated by SDS–PAGE for ~1 cm on a 4–12% Bis-Tris gel. The gel was Coomassie stained with SimplyBlue (Invitrogen) and each lane cut into two segments based on the ~70-kDa molecular weight marker. Gel pieces were de-stained in MilliQ water twice and dehydrated with NH_4_HCO_3_/50% acetonitrile (ACN) (20 min) and 100% ACN (2 × 5 min). Digestion was performed overnight at 37 °C with trypsin (20 ng µl^–1^ in 50 mM NH_4_HCO_3_). Peptides were extracted twice in 1% formic acid (FA)/50% ACN, once with 100% ACN and then dried to completion. The sample was then reconstituted in solvent A (2% ACN/0.1% FA) and injected onto a 0.1 × 100-mm^2^ Waters Symmetry 1.7-mm BEH-130 C18 column via an auto-sampler for separation by reverse-phase chromatography on a NanoAcquity UPLC system (Waters). A dual-stage linear gradient with a flow rate of 1 μl min^–1^ was applied for peptide separation, where solvent A was 0.1% FA/2% ACN/water and solvent B was 0.1% FA/2% water/ACN. Solvent B was increased from 2 to 25% over 35 min and then from 25 to 60% over 2 min, with a total analysis time of 60 min. Peptides eluting from the column were analyzed on an LTQ Orbitrap Elite mass spectrometer (Thermo Fisher) equipped with an Advance CaptiveSpray ionization source (Michrom). MS1 precursor ions were scanned in Fourier transform mass spectroscopy at 60,000 resolution and tandem mass spectrometry (MS/MS) data acquired on the 15 most abundant ions in LTQ linear ion trap mass spectrometer in data-dependent mode. MS/MS spectra were searched using the Mascot algorithm (Matrix Sciences) against a concatenated target-decoy database comprised of the FASTA sequences from the *N. naja* predicted proteome, Swiss-Prot entries from the Elapinae family (https://www.uniprot.org/taxonomy/42168) and common contaminant proteins, as well as the reversed versions of each sequence. Peptide spectral matches were filtered using a linear discriminant function to a peptide FDR of 5% to identify those that mapped to the toxin proteins identified from the genome.

### 3FTx structural modeling

Three-dimensional structural models were generated using MOE software (Chemical Computing Group, v.0101) and homology modeling was performed using the AMBER10 EHT forcefield^105^. Representative sequences were first searched in the protein database and the closest hits based on percentage sequence identity were chosen as templates for construction of structural homology models and for root mean square deviation calculations (Supplementary Table 10).

### Molecular evolutionary analysis

For pairwise comparisons of both venom-expressed and non-venom-expressed genes, the number of nucleotide substitutions per synonymous (*K*_S_) and non-synonymous site (*K*_A_) for each pair of protein-coding genes was computed according to the Nei–Gojobori method^106^ using Sqdif Plot (http://www.gen-info.osaka-u.ac.jp/~uhmin/study/sqdifPlot/index.html).

### Identification of ZRS limb enhancer deletion

To identify the ZRS enhancer region, sequences corresponding to this region were downloaded from ref. ^107^. BLASTn (v.2.2.29+)^99^ was used to identify the orthologous *N. naja* sequence. Multiple sequence alignment was performed using Clustal^108^.

### Variant analysis

We first performed phylogenetic analysis using 1,654 polymorphisms identified in the mitochondrial genomes using short-read whole-genome sequence data for each of the six study animals. Briefly, adapter and quality trimmed reads were aligned to the *N. naja* mitochondrial genome (GenBank: DQ343648.1) using BWA^93^. Variant calling was performed using SAMtools^109^. The resulting alignments of all six animals were compared to a reference^110^. Multiple sequence alignment of the consensus mitochondrial sequence from the study animals, generated using MUSCLE^108^, was used to construct the phylogenetic tree using FigTree v.4.2.

For the genome-wide protein-coding variant analysis, whole-genome sequencing data for the six study animals were mapped to the Nana_v5 reference genome assembly using BWA^93^ with default options. Mapped reads were sorted and duplicates marked with PicardTools. Local realignment, germline variant calling and joint genotyping were performed using GATK (v.4.1.0.0)^94,111^. Germline variants were decomposed and normalized with vt^112^ and functionally annotated with snpEff^113^ against the annotated genome. Germline variants that had genotype calls in all samples were used to calculate percentage identity values between samples.

### Genome heterozygosity estimation

K-mers from short-read sequencing data for each of the six animals used in this study were counted using Jellyfish (v.2.2.6)^114^ for several values of *K*: 15, 17 and 21. Each computed *K*-mer histogram was then analyzed with GenomeScope^115^.

### Reporting Summary

Further information on research design is available in the Nature Research Reporting Summary linked to this article.

## Online content

Any methods, additional references, Nature Research reporting summaries, source data, extended data, supplementary information, acknowledgements, peer review information; details of author contributions and competing interests; and statements of data and code availability are available at 10.1038/s41588-019-0559-8.

## Supplementary information

Supplementary Information Supplementary Note, Figs. 1–9

Reporting Summary

Supplementary Tables 1–12

## Data Availability

Sequence data (DNA and RNA) can be accessed at NCBI under BioProject accession no. PRJNA527614. The MS data are available under accession no. MSV000084564.

## Code availability

All software tools used in this study are provided in the accompanying Nature Research Reporting Summary document.
