# Supplementary material for: The Indian cobra reference genome and transcriptome enables comprehensive identification of venom toxins
Source: Nat Genet. 2020 Jan 6;52(1):106–17. doi: 10.1038/s41588-019-0559-8 (PMC8075977; doi:10.1038/s41588-019-0559-8)
Supplement: Supplementary file 1 — Supplementary Note, Figs. 1–9 [file 41588_2019_559_MOESM1_ESM.pdf]

nature genetics

SUPPLEMENTARY INFORMATION

https://doi.org/10.1038/s41588-019-0559-8

In the format provided by the authors and unedited.

The Indian cobra reference genome and transcriptome enables comprehensive identification of venom toxins

Kushal Suryamohan[1,2], Sajesh P. Krishnankutty[3,4], Joseph Guillory[1], Matthew Jevit[5], Markus S. Schröder[1], Meng Wu[1], Boney Kuriakose[3], Oommen K. Mathew[3], Rajadurai C. Perumal[3], Ivan Koludarov[6], Leonard D. Goldstein[1,7], Kate Senger[1], Mandumpala Davis Dixon[3], Dinesh Velayutham[3], Derek Vargas[1,2], Subhra Chaudhuri[1], Megha Muraleedharan[3], Ridhi Goel[3], Ying-Jiun J. Chen[1], Aakrosh Ratan[8], Peter Liu[9], Brendan Faherty[9], Guillermo de la Rosa[10], Hiroki Shibata[11], Miriam Baca[12], Meredith Sagolla[12], James Ziai[12], Gus A. Wright[13], Domagoj Vucic[14], Sangeetha Mohan[15], Aju Antony[15], Jeremy Stinson[1], Donald S. Kirkpatrick[9], Rami N. Hannoush[14], Steffen Durinck[1,7], Zora Modrusan[1], Eric W. Stawiski[1,2], Kristen Wiley[16], Terje Raudsepp[5], R. Manjunatha Kini[17], Arun Zachariah[4,18] and Somasekar Seshagiri[1,4]*

[1]Molecular Biology Department, Genentech, Inc., South San Francisco, CA, USA. [2]MedGenome Inc., Foster City, CA, USA. [3]AgriGenome Labs Private Ltd, Kochi, India. [4]SciGenom Research Foundation, Bangalore, India. [5]Molecular Cytogenetics laboratory, Texas A&M University, College Station, TX, USA. [6]Ecology and Evolution Unit, Okinawa Institute of Science and Technology, Onna-son, Japan. [7]Department of Bioinformatics and Computational Biology, Genentech, Inc., South San Francisco, CA, USA. [8]Center for Public Health Genomics, University of Virginia, Charlottesville, VA, USA. [9]Department of Microchemistry Proteomics, and Lipidomics, Genentech, Inc., South San Francisco, CA, USA. [10]The Donnelly Centre for Cellular and Biomolecular Research, University of Toronto, Toronto, Ontario, Canada. [11]Division of Genomics, Medical Institute of Bioregulation, Kyushu University, Fukuoka, Japan. [12]Department of Pathology, Genentech, Inc., South San Francisco, CA, USA. [13]College of Veterinary Medicine, Flow Cytometry Shared Resource Laboratory, Texas A&M University, College Station, TX, USA. [14]Department of Early Discovery Biochemistry, Genentech, Inc., South San Francisco, CA, USA. [15]Department of Molecular Biology, SciGenom Labs, Kochi, India. [16]Kentucky Reptile Zoo, Slade, KY, USA. [17]Department of Biological Sciences, National University of Singapore, Singapore, Singapore. [18]Wayanad Wildlife Sanctuary, Sultan Bathery, India. *e-mail: sekar@sgrf.org

The Indian cobra reference genome and transcriptome enables comprehensive identification of venom toxins

Kushal Suryamohan[1,2], Sajesh P. Krishnankutty[3,4], Joseph Guillory[1], Matthew Jevit[5], Markus Schroeder[1], Meng Wu[1], Boney Kuriakose[3], Oommen K. Mathew[3], Rajadurai C. Perumal[3], Ivan Koludarov[6], Leonard D. Goldstein[1,7], Kate Senger[1], Mandumpala Davis Dixon[3], Dinesh Velayutham[3], Derek Vargas[1], Subhra Chaudhuri[1], Megha Muraleedharan[3], Ridhi Goel[3], Ying-Jiun J. Chen[1], Aakrosh Ratan[8], Peter Liu[9], Brendan Faherty[9], Guillermo de la Rosa[10], Hiroki Shibata[11], Miriam Baca[12], Meredith Sagolla[12], James Ziai[12], Gus A.Wright[13], Domagoj Vucic[14], Jeremy Stinson[1], Donald S. Kirkpatrick[9], Rami N. Hannoush[14], Steffen Durinck[1,7], Zora Modrusan[1], Eric W. Stawiski[1,2], Kristen Wiley[15], Terje Raudsepp[5], R. Manjunatha Kini[16], Arun Zachariah[4,17], Somasekar Seshagiri[1,4,*]

[1]Molecular Biology Department, Genentech, Inc., South San Francisco, CA, USA. [2]Medgenome Inc., Foster city, CA, USA. [3]AgriGenome Labs Private Limited, Kakkanad, Kochi, Kerala, India. [4]SciGenom Research Foundation, Bangalore, Karnataka, India. [5]Molecular Cytogenetics laboratory, Texas A&M University, College Station, TX, USA. [6]Ecology and Evolution Unit, Okinawa Institute of Science and Technology, Onna, Kunigami-gun, Okinawa, Japan. [7]Department of Bioinformatics and Computational Biology, Genentech, Inc., South San Francisco, CA, USA. [8]Center for Public Health Genomics, University of Virginia, Charlottesville, VA, USA. [9]Department of Microchemistry, Proteomics, and Lipidomics, Genentech, Inc., South San Francisco, CA, USA. [10]The Donnelly Centre for Cellular and Biomolecular Research, University of Toronto, Toronto, ON, Canada. [11]Division of Genomics, Medical Institute of Bioregulation, Kyushu University, Fukuoka, Japan. [12]Department of Pathology, Genentech, Inc., South San Francisco, CA, USA. [13]College of Veterinary Medicine, Flow Cytometry Shared Resource Laboratory, Texas A&M University, College Station, TX USA. [14]Department of Early Discovery Biochemistry, Genentech, Inc., South San Francisco, CA, USA. [15]Kentucky Reptile Zoo, Slade, KY, USA. [16]Department of Biological Sciences, National University of Singapore, Singapore. [17]Wayanad wildlife sanctuary, Sultan Bathery, Kerala, India.

[*]Correspondence: S.S., sekar@sgrf.org; Phone: 650-539-2458

Table of contents

		Page #
1.	Supplementary Results	
1.1.	Genome Assembly	3
1.2.	10x genome assembly	4
1.3.	Chromosome assignment	5
1.4.	Sex chromosome scaffold identification	6
1.5.	Genome features	6
1.6.	Genome annotation	7
1.7.	Differential gene expression analysis	9
1.8.	Indian cobra toxin genes	10
1.8.1.	Phospholipase A2 genes	10
1.8.2.	Phospholipase B genes	10
1.8.3.	Acetylcholinesterase genes	10
1.9.	Genetic diversity	11
2.	References	13
3.	Supplementary Figures	16
4.	List of Supplementary Tables	30

1. Supplementary Results

1.1. Genome assembly

Canu (v1.6)[1] was used to combine the Pacbio and Oxford nanopore reads to generate the initial draft assembly that consisted of 13,066 contigs with an N50 of 310 kb and a total length of 1.66 Gb. Next, Illumina reads (2 x 150 bp) were aligned to this initial assembly using BWA (v0.7.10-r789) and SAMtools (v1.2). The resulting alignment was then used as input to Pilon (v1.22)[2] for error correction and conflict resolution to produce a draft assembly Nana_v1 (Fig. 1a, Supplementary Fig. 3 and Table 1). Pilon identified 7,781,521 bases with equal-probability heterozygous substitutions, indicating potential variant sites within the genome. Five iterative rounds of Pilon correction were performed to lower base call errors and improve gene completeness. After Pilon correction, BUSCO gene completeness (tetrapoda lineage) in Nana_v1 improved from 60.5% (41.5% complete, 19% fragmented) to 94.3% (86.7% complete, 6.7% fragmented) (Supplementary Fig. 3). We also tested other assembly algorithms such as Flye[3] (46,191 contigs, 0.06 Mb contig N50) and WTDBG2 (https://github.com/ruanjue/wtdbg2) (15,686 contigs, 0.238 contig N50) and found that Canu generated the most contiguous assembly. The polished Nana_v1 assembly and Chicago[4] library reads were then used as input data for HiRise, a software pipeline designed specifically for using proximity ligation data to scaffold genome assemblies (Dovetail Genomics). Briefly, Chicago library sequences were aligned to the draft input assembly using a modified SNAP (v0.15.4) read mapper (http://snap.cs.berkeley.edu). The separations of Chicago read pairs mapped within draft contigs were analyzed by HiRise to produce a likelihood model for genomic distance between read pairs, and the model was used to identify and break putative misjoins, to score prospective joins, and make joins to produce an intermediate genome assembly, Nana_v2, with 2,647 scaffolds and a scaffold N50 of 4.855 Mb (Fig. 1b, Table 1 and Supplementary Table 2a). Next, a second round of scaffolding was performed using the BNG optical mapping data. Bionano Access (v1.3.0) (BioNano Genomics, USA) software application was used to

combine the optical map data (Nana_v3) with Nana_v2 to produce a scaffolded assembly, Nana_v4 that consisted of 2,167 scaffolds (scaffold N50 of 143.3 Mb) (Fig. 1c and Supplementary Table 2b). Nana_v4 contained 95.8% of Nana_v2 (corresponding to 88 scaffolds) assembly. To test if the order of integration of BNG or Chicago data impacts the contiguity of the assembly, we also tried to scaffold Nana_v1 with BNG data. This resulted in a 2.11 Gb genome assembly that contained 9,505 scaffolds (scaffold N50 of 71.68 Mb). However, this assembly had gaps that spanned ~300 Mb (14.2%). By contrast, only ~6% of Nana_v4 contained gaps. This demonstrated that Chicago data is more effective at intermediate range scaffolding and we decided to use Nana_v2 for scaffolding with BNG data. Finally, Hi-C library sequences were aligned and scaffolded following the same method as with the Chicago data using HiRise (Dovetail Genomics, USA). This resulted in a final assembly containing 1,911 scaffolds (Nana_v5; Supplementary Fig. 4) that had a scaffold N50 of 223.35 Mb (1.4x improvement; Fig. 1d and Supplementary Table 2c). The largest scaffold from Nana_v5 assembly was 375 Mb. Approximately 6.4% of Nana_v5 assembly contained gaps. We then filtered 14 scaffolds from this assembly that contained contaminant (viral, bacterial, plasmid, archaea, and human) or mitochondrial sequences using Kraken[5]. A majority of the remaining scaffolds (1,511 scaffolds) were highly repetitive (>75% of each scaffold was comprised of repetitive elements). Although the Nana_v5 assembly consisted of 1,897 scaffolds, ~95% of the genome was contained within 19 scaffolds that were >10 Mb. The assembly strategy we used in this study resulted in improved contiguity at each successive step as the indicated genomic data type was added, resulting in the most contiguous reptilian genome assembly generated to date (Table 1). Consistent with the high contiguity of this genome, analysis of complete near-universal single-copy orthologs using BUSCO[6] based on core metazoan gene models estimated a 94% genome completeness for the Indian cobra assembly.

1.2. 10x Genome assembly

Using 10x genomics technology (see methods), a total of 761.34 million linked-reads (114 Gb, 2 x 150 bp) with a raw coverage of ~65x were generated for a female Indian cobra (NN05). Molecule size following sequencing of the Chromium library was estimated to be 63.81 kb. The linked-read data was assembled using the Supernova^{TM} assembler (v2.x)[7]. This resulted in a diploid assembly with locally phased haplotype blocks or 'pseudohaps'. A single pseudohap assembly of 2.061 Gb assembly consisted of 48,370 scaffolds with a contig N50 of 66 kb, scaffold N50 of 42.41 Mb, and phase block N50 of 41.45 Mb (Supplementary Fig. 5). A total of 58.1% of the assembly was phased with a largest scaffold of 145.44 Mb. We then combined this assembly with BNG optical map data for NN05 that resulted in a 2.11 Gb assembly consisting of 48,183 scaffolds and an improved scaffold N50 of 147.3 Mb. This 10x-BNG assembly contained 236 scaffolds from the primary 10x assembly and the largest scaffold was 305 Mb with about 85% of the entire assembly contained within 29 scaffolds >10 Mb. However, this assembly included 48,134 unplaced scaffolds (~0.31 Gb). Pairwise alignment of the 10x-BNG and Nana_v5 assembly revealed that the 10x-BNG assembly was more fragmented than Nana_v5 with multiple scaffolds corresponding to individual Nana_v5 scaffolds (Supplementary Fig. 5c and 5d). These redundant scaffolds from the 10x-BNG assembly likely resulted in the increased assembled genome size estimate observed with the synthetic long-read assembly. Further, 18% of the 10x-BNG assembly contained gaps compared to the estimated genome size.

1.3. Chromosome Assignment

In total, we were able to assign four Nana_v5 scaffolds to four *El. quad* MACs, that included three entire chromosomes (1, 3, and Z), and five scaffolds to MICs (Fig. 1e, Supplementary Tables 3a and 3b)[8]. Additionally, we used single chromosome sequencing (SChrom-seq; see methods) data to confirm the chromosomal assignment for 6 MACs. We then assigned the remaining 8 scaffolds (>10 Mb) to 2 MACs and 6 MICs

in the order of decreasing scaffold length, per convention (Figs. 1f, 2a, Supplementary Tables 3b and 3c).

1.4. Sex chromosome scaffold identification

We utilized cDNA markers from the distant *El. quad* and SChrom-seq data to identify a single Z-linked scaffold, representing ~154 Mb (Supplementary Tables 3a, 3b and 3c) in the Nana_v5 assembly. To identify the W-linked scaffolds we separately aligned Illumina reads obtained from NN05 (female) and NN04 (male) to the 10x-BNG hybrid NN05 assembly using BWA[9] allowing for two mismatches and one indel. Scaffolds with less than 80% alignment coverage were excluded from further analysis. Then, single-base depths were calculated using SAMtools[10] following which coverage and mean depth for each scaffold was calculated. Using the average coverage across a scaffold using either the male or female reads, we identified a 52.1Mb W-linked scaffold (Super_scaffold_1000010; Fig. 2a and Supplementary Table 3d). We confirmed this scaffold to be W-linked by searching for the W-linked gametolog *CTNNB1* (Catenin Beta 1)[11].

1.5. Genome Features

Overall, the Indian cobra genome was 43.22% (~760 Mb) repetitive with long interspersed elements (LINEs; ~9% of genome) and long terminal retrotransposons (LTRs; ~8% of genome) being the major classes of transposable elements (TEs) in the genome (Supplementary Table 4a). Comparison of repeat element distribution in the Indian cobra genome with king cobra, boa, python, five-pace viper, prairie rattlesnake and lizard genomes revealed that the fraction of LTRs in the Indian cobra genome was higher (Extended Data Fig. 3 and Supplementary Table 4b).

1.6. Genome annotation

Gene prediction was performed on Nana_v5 using MAKER (v2.31.10)[12,13] in an iterative process. First, *ab initio* gene prediction was performed by the programs SNAP (v2006-07-28)[14] and Augustus (v3.2.3)[15] using the multi-tissue *N. naja* transcriptome assembly (Supplementary Table 1a) and a protein database that combined the UniProt/Swiss-Prot and NCBI non-redundant database of reviewed reptilian proteins. A total of three iterative runs of MAKER was used to refine the gene models and produce the final gene set with an annotation edit distance (AED) cutoff of 0.5[12,13]. Genome annotation quality was assessed by BUSCO analysis using the conserved core set of metazoan (94% BUSCO score) and tetrapod genes (85% BUSCO score). Using the set of complete predicted *N. naja* protein sequences, we developed a functional annotation pipeline that used InterProScan (v5.30-69.0)[16,17] and BLASTp (v2.2.29+)[18] to search for orthologs in the closest species available in the NCBI HomoloGene database (*Gallus gallus*; https://www.ncbi.nlm.nih.gov/homologene). The predicted protein sequences were also queried against the human proteome. Best-matching orthologs from *N. naja* were assigned the corresponding human gene symbols using HuGo gene nomenclature committee (HGNC). Further, UniProt and TrEMBL protein databases were also utilized for improving the protein annotation. BLASTp was performed by using default search parameters. Hits with a query coverage >70% and percentage identity >80% were considered as the best hit. To further improve the protein annotation, each predicted protein was compared with conserved domain/motif level protein information using Pfam, CDD and COG databases for higher-level protein family classification (Supplementary Tables 5 and 7b).

To annotate the venom gland toxin genes, we used a combination of full-length iso-seq (Pacbio) venom gland transcriptome data, gene sequences from 38 venom gene families in GenBank and the curated toxin genes in the Tox-Prot database[19]. We first searched our full-length transcriptome data using tBLASTx against the known venom genes (e-value cutoff of 1-e05). For each candidate venom gene transcript hit, we performed a reciprocal tBLASTx to confirm its identity as a venom gene. The three most

abundant venom gene families at the transcriptome level and number of genes were three finger toxins (3FTxs), snake venom metalloproteinases (SVMPs), and cysteine rich secretory proteins (CRISPs) (Supplementary Table 6c). Further, existing curated protein sequence data from the Tox-prot database[19] was used to search the genome and transcriptome for toxin genes that were not annotated by the process described above or by MAKER via BLAST (v2.2.29+)[18]. Additional curation was performed using Exonerate[20] to establish the intron–exon boundaries of the manually identified toxin genes. For 3FTx gene annotation, we combined HMM-based gene prediction together with the manual curation to identify 14 3FTx genes while the remaining genes were identified by using a modified version of a previously described method[21]. Further manual curation was performed for 83 full-length hypothetical/unknown genes via BLASTp (v2.2.29+)[18] with the "automatic parameter adjust option" set for short length queries. The results were then manually checked against the conserved domain database (CDD) hits to identify 12 putative toxin genes. In total, 23,248 protein coding genes, and 31,447 transcripts were annotated in the genome including 139 genes that were curated as a toxin gene in the Tox-prot database. A mean 9 ± 1 exons per gene, with an average size of 279 bp, was predicted. The average intron length observed was 1991 bp. The mean transcript length we observed was 2677 bp and the mean CDS length was 1389 bp. The W-linked scaffold from the 10x-BNG hybrid assembly was annotated in a similar manner to identify 284 genes (437 transcripts) with an average intron length of 1864 bp, a mean transcript length of 2319 bp and a mean CDS length of 1253 bp.

King cobra and lizard orthologs were identified using BLASTp (v2.2.29+)[18] using a number of different search parameters. Using default BLASTp parameters, all Indian cobra predicted proteins were queried against the curated king cobra and lizard proteomes. Stringent filtering was then performed using a minimum query coverage of >=70% with >= 80% identity to identify a high-confidence set of orthologs.

To identify venom gene orthologs between the king cobra and Indian cobra, all Indian cobra venom gland toxin protein sequences from Supplementary Table 6b were

provided as input along with all annotated king cobra proteins to OrthoFinder (v2.3.1)[22] (Supplementary Table 6e). Clustering was performed by setting the "-M" flag to run in msa (multiple sequence alignment) mode with "-S" diamond sequence search method. The target coverage value for gene match was set with default parameters.

As an additional quality check of our genome annotation, we searched for the *Hox* gene complex in *N. naja.* We annotated 37 *Hox* genes in the *N. naja* genome and it did not include the *Hoxd12* gene, involved in limb development in tetrapods[23]. Further, we found a deletion in a distal enhancer element of the *Sonic hedgehog* gene that previously has been attributed to loss of limbs in snakes including the king cobra, boa, speckled rattlesnake, python and the European viper (Supplementary Fig. 6)[24].

Using the predicted protein-coding genes, we identified several key signaling pathway components to be conserved in the Indian cobra genome, including the Hedgehog, WNT, receptor tyrosine kinase, immune response and cell death pathways (Supplementary Table 11). Members of the WNT signaling pathway are involved in stem cell maintenance and regeneration[25,26]. We found expression of several WNT pathway genes including the Wnt ligands (*Nana08381*, and *Nana10175*), Frizzed (*Nana16725*, *Nana21441*, *Nana22930*, *Nana25367*, and *Nana35345*), and LRP (*Nana04287*, and *Nana04290*) in the venom glands (Supplementary Fig. 7). They likely play a key role in venom gland biogenesis, maintenance and renewal.

1.7. Differential gene expression analysis

Overall, we observed tissue-specific genes that were upregulated in the liver (n=241), kidney (n=132), venom gland (n=109), lung (n=34), pancreas (n=60) and ovary (n=44) (Extended Data Fig. 5, Supplementary Tables 7c and 7h). Differentially upregulated genes (DUGs) in the liver included peptidase S1, serpin, fetuin, sulfotransferase, lipocalin, lipase, and cytochrome P450 genes, while top gene ontology (GO) term hits (Benjamini-Hochberg adjusted p-value <0.005) included serine-type endopeptidase activity, cysteine-type endopeptidase activity, iron ion binding, lipase activity, protease

inhibition activity, sulfotransferase activity, fatty-acid metabolism and hyaluronan metabolism. Kidney-specific DUGs encoded transmembrane transporter proteins such as solute carrier genes (SLC group), as well as mitochondrial carrier genes. The top GO terms for the kidney (Benjamini-Hochberg adjusted p-value <0.005) included anion transport, acid secretion and potassium ion import. GO terms enriched in the pancreas (Benjamini-Hochberg adjusted p-value <0.005) included regulation of glucokinase activity and metabolism.

1.8. Indian Cobra toxin genes

1.8.1. Phospholipase A2 (PLA2) genes

It is important to note that both PLA2s (*Nana39244* and *Nana39246*) identified in this study contained conserved Tyr27, Gly31 and Asp48 residues close to the calcium binding loop (Gly30, Asp49) as well as residues Gly29, His47, Tyr51, Tyr67 and Asp94, crucial for enzymatic activity[27] (Supplementary Fig. 2). The presence of these conserved residues strongly suggests that these proteins might contribute to the neurotoxic effect of *N. naja* venom[28].

1.8.2. Phospholipase B genes

Phospholipase B (PLB) is a minor component of snake venom that have hemolytic activity [29]. About 20 PLB sequences mostly derived from transcriptomic or genomic data have been reported[30,31]. In the *N. naja* genome we identified two PLBs sequences that previously have not reported and they likely contribute to the hemolytic properties of *N. naja* venom. The two PLBs encoded by *Nana27183* and *Nana35026* shared 93% and 95% identity to PLB from *Notechis scutatus* and *Pseudonaja textilis*. Further, we detected two full-length c-type natriuretic peptide genes, *Nana20849* and *Nana20852,* in the venom gland transcriptome. Nana20852 showed a venom gland specific expression and shared high homology (93% identity) with UniProtKB: D9IX97.1, a potent hypotensive natriuretic peptide from *Naja atra*[32].

1.7.3. Acetylcholinesterase genes

We detected 17 Type-B carboxylesterase genes including Acetylcholinesterase (AChE), encoded by *Nana38737*, as well as several lipases. AChEs are members of the cholinesterase family[33] that play a vital role in acetylcholine transmission in the nervous system where they hydrolyze acetylcholine to choline and acetate, thereby terminating the chemical impulse. AChE expression has previously been reported in snake venom, particularly in Elapidae, with the exception of *Dendroaspis* species[34]. However, outside of the cholinergic system, the role of venom AChEs remains to be characterized.

1.9. Genetic Diversity

Overall, the heterozygosity of the cobras from India was ~0.9% while the those from the zoo in Kentucky were ~0.8% (see methods). To understand the genetic polymorphisms in our study animals, we first compared the mitochondrial genomes and found that the six individuals were not maternally related. A total of 1,654 mitochondrial SNPs identified across the 6 study animals were used to construct a phylogenetic tree (Supplementary Fig. 8). We observed that 3 of the study animals from the Kentucky reptile zoo (NN04, NN05, NN06) were more closely related to each other than to those from India (NN01, NN02). The number of SNPs in the study animals ranged from 33-152 and NN03 had the most divergent mitochondrial genome compared to the other study animals with an average of 620 pair-wise SNPs (Supplementary Fig. 8).

Next, we assessed the extent of genome-wide non-synonymous polymorphisms in all protein-coding genes among the study animals. We computed pair-wise genotype similarity estimates (PWS) (see materials and methods) between the six study animals (Extended Data Fig. 10). Overall, we observed a pair-wise genotype similarity estimates (PWS) ranging from 45.9 to 63.3%; (Extended Data Fig. 10 and Supplementary Table 12a). Venom gland-expressed genes had a PWS in the range of 44.6-63.7% (Extended Data Fig. 10b, 10e and Supplementary Table 12b), comparable to the entire proteome. However, the PWS for genes expressed only in the venom gland ranged from 41.8-70.2% (Extended Data Fig. 10c and Supplementary Table 12c). Interestingly, we

observed the greatest PWS score variability for the 19 3FTx and it ranged from 31-80.6% (Extended Data Fig. 10d, Supplementary Fig. 9a-s and Supplementary Table 12d). These findings indicate that non-synonymous substitutions in toxin genes can contribute to variable antivenom efficacy and needs to be considered when developing antivenom.

References

1. Koren, S. *et al.* Canu: scalable and accurate long-read assembly via adaptive k-mer weighting and repeat separation. *Genome Res* **27**, 722-736 (2017).
2. Walker, B.J. *et al.* Pilon: an integrated tool for comprehensive microbial variant detection and genome assembly improvement. *PLoS One* **9**, e112963 (2014).
3. Kolmogorov, M., Yuan, J., Lin, Y. & Pevzner, P.A. Assembly of long, error-prone reads using repeat graphs. *Nat Biotechnol* **37**, 540-546 (2019).
4. Putnam, N.H. *et al.* Chromosome-scale shotgun assembly using an in vitro method for long-range linkage. *Genome Res* **26**, 342-50 (2016).
5. Wood, D.E. & Salzberg, S.L. Kraken: ultrafast metagenomic sequence classification using exact alignments. *Genome Biol* **15**, R46 (2014).
6. Simao, F.A., Waterhouse, R.M., Ioannidis, P., Kriventseva, E.V. & Zdobnov, E.M. BUSCO: assessing genome assembly and annotation completeness with single-copy orthologs. *Bioinformatics* **31**, 3210-2 (2015).
7. Weisenfeld, N.I., Kumar, V., Shah, P., Church, D.M. & Jaffe, D.B. Direct determination of diploid genome sequences. *Genome Res* **27**, 757-767 (2017).
8. Matsubara, K. *et al.* Evidence for different origin of sex chromosomes in snakes, birds, and mammals and step-wise differentiation of snake sex chromosomes. *Proc Natl Acad Sci U S A* **103**, 18190-5 (2006).
9. Li, H. & Durbin, R. Fast and accurate short read alignment with Burrows-Wheeler transform. *Bioinformatics* **25**, 1754-60 (2009).
10. Li, H. *et al.* The Sequence Alignment/Map format and SAMtools. *Bioinformatics* **25**, 2078-9 (2009).
11. Vicoso, B., Emerson, J.J., Zektser, Y., Mahajan, S. & Bachtrog, D. Comparative sex chromosome genomics in snakes: differentiation, evolutionary strata, and lack of global dosage compensation. *PLoS Biol* **11**, e1001643 (2013).
12. Campbell, M.S., Holt, C., Moore, B. & Yandell, M. Genome Annotation and Curation Using MAKER and MAKER-P. *Curr Protoc Bioinformatics* **48**, 4 11 1-39 (2014).
13. Holt, C. & Yandell, M. MAKER2: an annotation pipeline and genome-database management tool for second-generation genome projects. *BMC Bioinformatics* **12**, 491 (2011).
14. Korf, I. Gene finding in novel genomes. *BMC Bioinformatics* **5**, 59 (2004).
15. Stanke, M., Schoffmann, O., Morgenstern, B. & Waack, S. Gene prediction in eukaryotes with a generalized hidden Markov model that uses hints from external sources. *BMC Bioinformatics* **7**, 62 (2006).
16. Jones, P. *et al.* InterProScan 5: genome-scale protein function classification. *Bioinformatics* **30**, 1236-40 (2014).

17. Mulder, N. & Apweiler, R. InterPro and InterProScan: tools for protein sequence classification and comparison. *Methods Mol Biol* **396**, 59-70 (2007).
18. Altschul, S.F., Gish, W., Miller, W., Myers, E.W. & Lipman, D.J. Basic local alignment search tool. *J Mol Biol* **215**, 403-10 (1990).
19. Jungo, F., Bougueleret, L., Xenarios, I. & Poux, S. The UniProtKB/Swiss-Prot Tox-Prot program: A central hub of integrated venom protein data. *Toxicon* **60**, 551-7 (2012).
20. Slater, G.S. & Birney, E. Automated generation of heuristics for biological sequence comparison. *BMC Bioinformatics* **6**, 31 (2005).
21. Koludarov, I. & Aird, S.D. Snake venom NAD glycohydrolases: primary structures, genomic location, and gene structure. *PeerJ* **7**, e6154 (2019).
22. Emms, D.M. & Kelly, S. OrthoFinder: solving fundamental biases in whole genome comparisons dramatically improves orthogroup inference accuracy. *Genome Biol* **16**, 157 (2015).
23. Zakany, J., Kmita, M. & Duboule, D. A dual role for Hox genes in limb anterior-posterior asymmetry. *Science* **304**, 1669-72 (2004).
24. Kvon, E.Z. *et al.* Progressive Loss of Function in a Limb Enhancer during Snake Evolution. *Cell* **167**, 633-642 e11 (2016).
25. Clevers, H., Loh, K.M. & Nusse, R. Stem cell signaling. An integral program for tissue renewal and regeneration: Wnt signaling and stem cell control. *Science* **346**, 1248012 (2014).
26. Steinhart, Z. & Angers, S. Wnt signaling in development and tissue homeostasis. *Development* **145**(2018).
27. Lambeau, G. *et al.* Structural elements of secretory phospholipases A2 involved in the binding to M-type receptors. *J Biol Chem* **270**, 5534-40 (1995).
28. Sribar, J., Oberckal, J. & Krizaj, I. Understanding the molecular mechanism underlying the presynaptic toxicity of secreted phospholipases A(2): an update. *Toxicon* **89**, 9-16 (2014).
29. Aloulou, A., Rahier, R., Arhab, Y., Noiriel, A. & Abousalham, A. Phospholipases: An Overview. *Methods Mol Biol* **1835**, 69-105 (2018).
30. Rokyta, D.R., Lemmon, A.R., Margres, M.J. & Aronow, K. The venom-gland transcriptome of the eastern diamondback rattlesnake (Crotalus adamanteus). *BMC Genomics* **13**, 312 (2012).
31. Margres, M.J., Aronow, K., Loyacano, J. & Rokyta, D.R. The venom-gland transcriptome of the eastern coral snake (Micrurus fulvius) reveals high venom complexity in the intragenomic evolution of venoms. *BMC Genomics* **14**, 531 (2013).
32. Zhang, Y. *et al.* A novel natriuretic peptide from the cobra venom. *Toxicon* **57**, 134-40 (2011).

33. Frobert, Y. *et al.* Acetylcholinesterases from Elapidae snake venoms: biochemical, immunological and enzymatic characterization. *Biochim Biophys Acta* **1339**, 253-67 (1997).

34. Kumar, V. & Elliott, W.B. The acetylcholinesterase of Bungarus fasciatus venom. *Eur J Biochem* **34**, 586-92 (1973).

2. Supplementary Figures

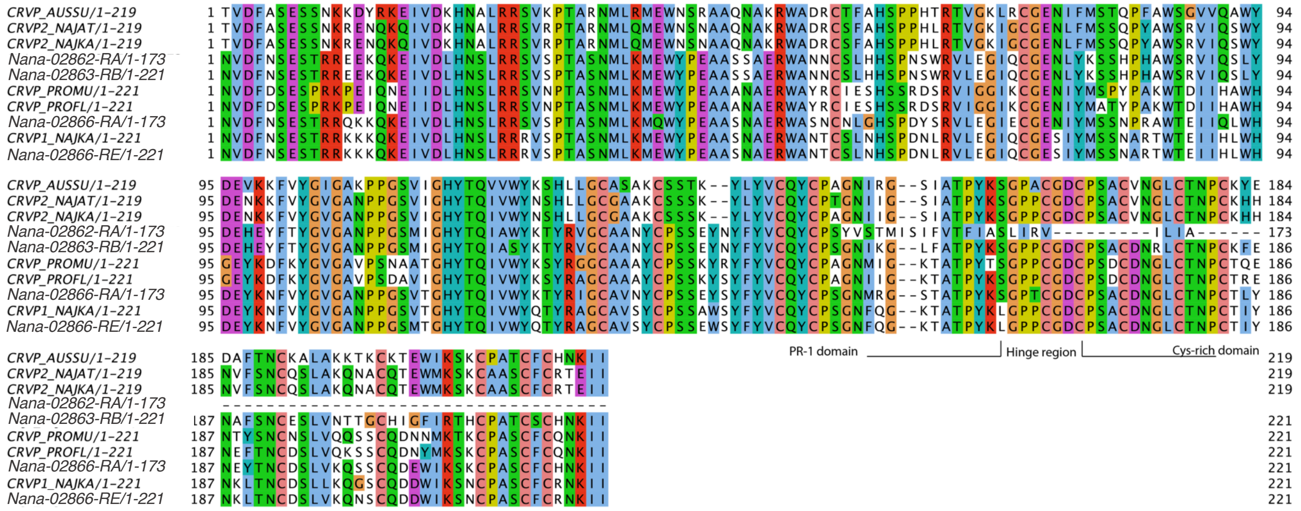

Supplementary Figure 1. CRISP expression and comparative sequence protein alignment. Multiple sequence alignment of CRISP proteins from the Indian cobra, other elapids and viperids. CRISP – cysteine-rich secretory protein; Elapid species – AUSS, *Austrelaps superbus*; NAJAT, *Naja atra*; NAJKA, *Naja kaouthia*; OPHHA, *Ophiophagus hannah;* Viperid species – PROFL, *Protobothrops flavoviridis*

a

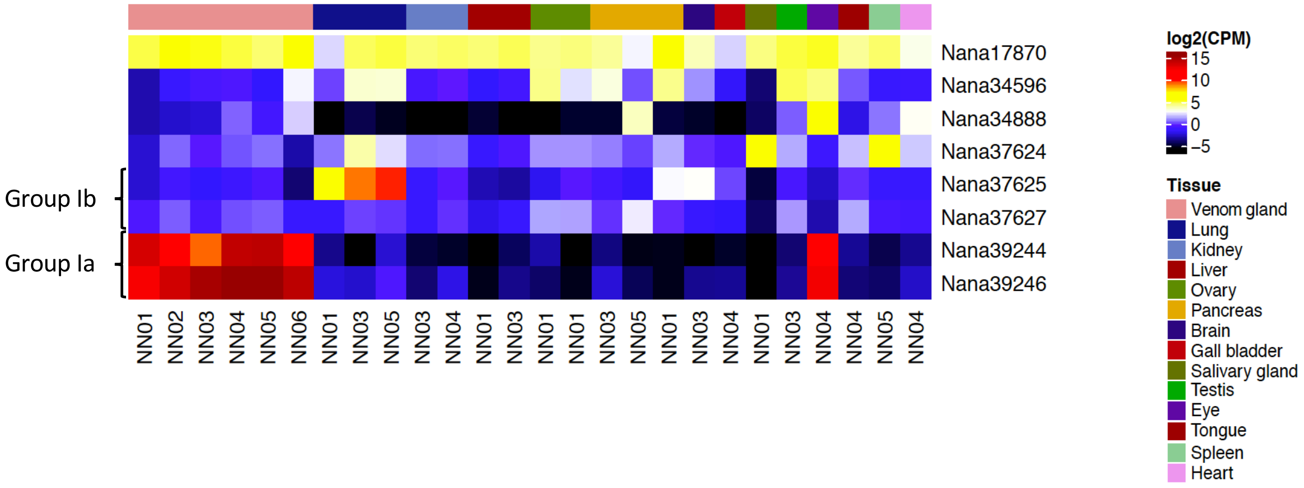

b

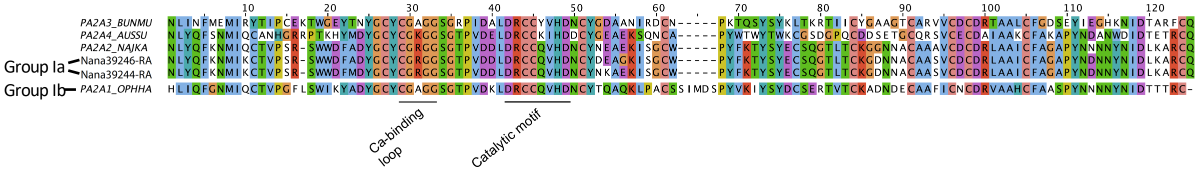

Supplementary Figure 2. PLA2 expression and comparative protein sequence alignment. (**a**), Heatmap representation of the expression of secretory Phospholipase A2 (PLA2) genes present in the India cobra genome. (**b**), Multiple sequence alignment of group I PLA2 proteins from the Indian cobra and other indicated elapids. (**c**), Two group II PLA2 proteins from the Indian cobra aligned to homologous proteins from representative viperid species. Elapid species – BUNMU, *Bungarus multicinctus*; AUSS, *Austrelaps superbus*; OPHHA, *Ophiophagus hannah;* NAJKA, *Naja kaouthia.* Expression values plotted as log_2 transformed CPM values.

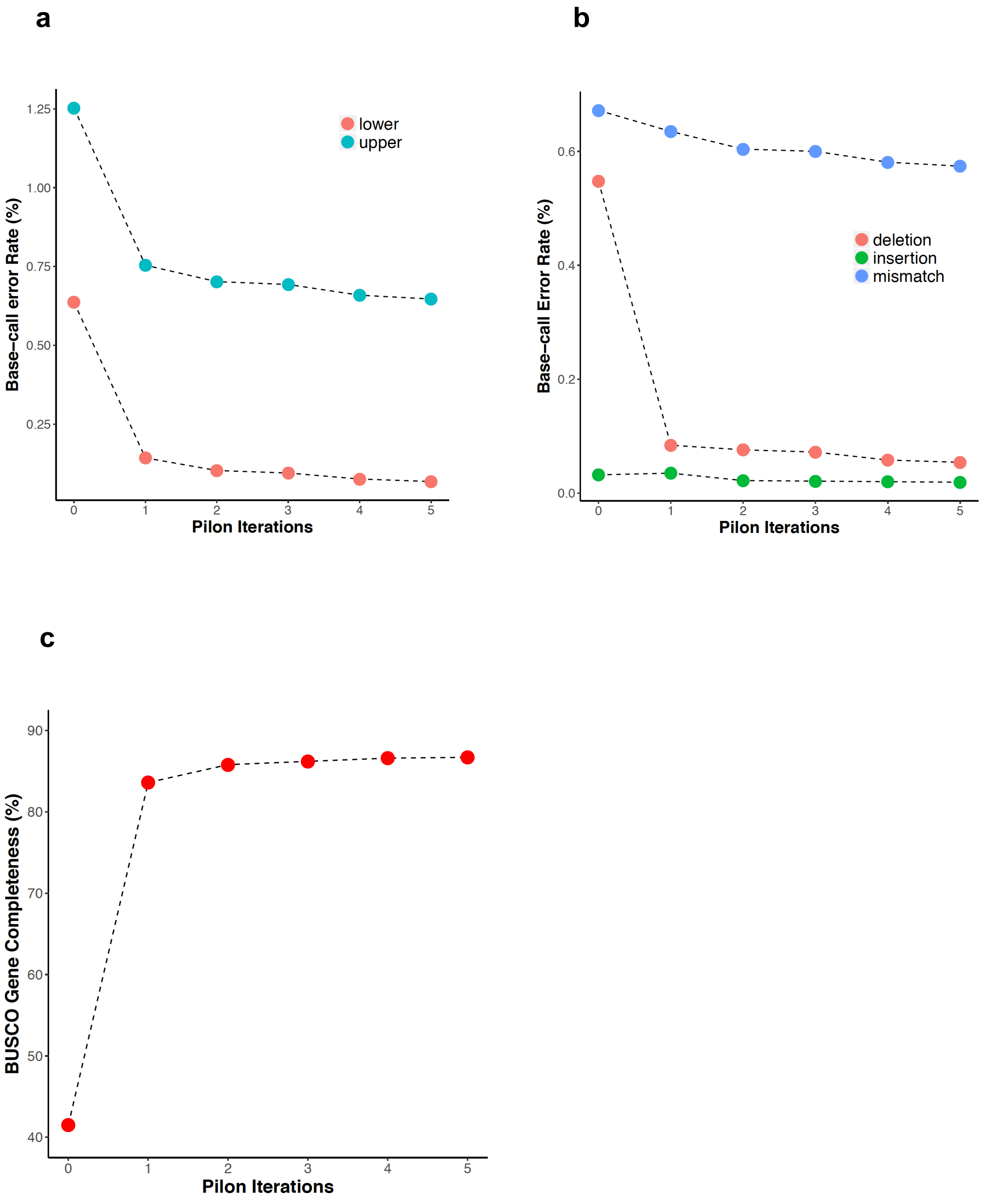

Supplementary Figure 3. Pilon improves base call accuracy and gene completeness. Five iterations of Pilon were used to polish the long-read assembly (Nana_v1), correct the assembly (**a**, **b**) and (**c**) improve gene completeness as assessed by BUSCO.

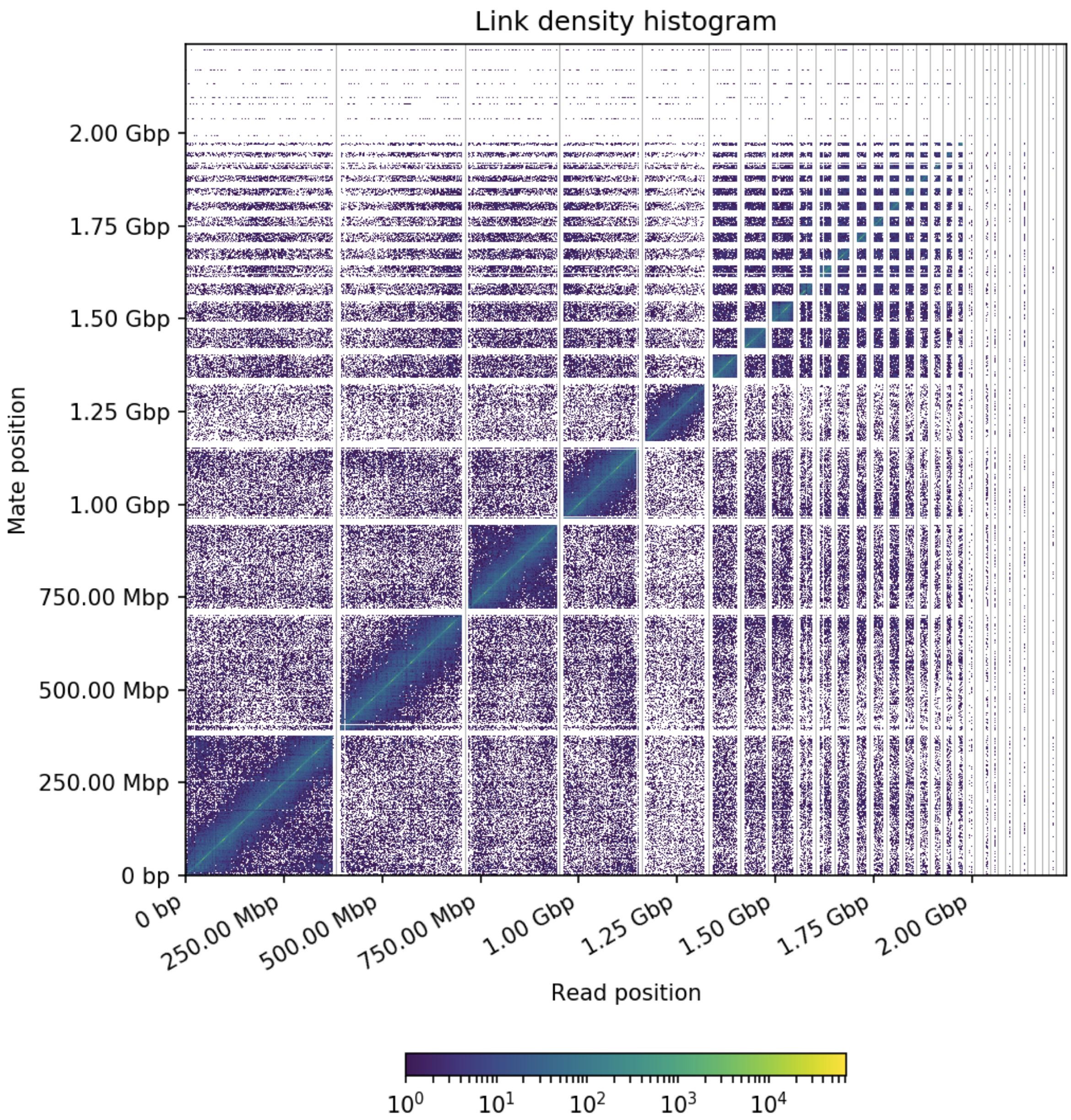

Supplementary Figure 4. Hi-C linkage map showing density of contacts.

a

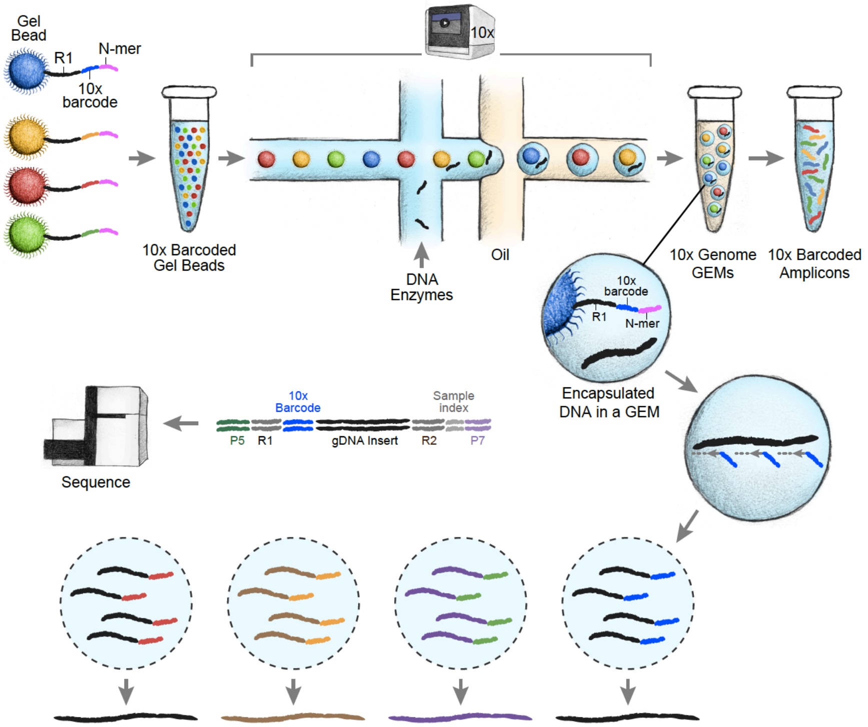

b

Assembly	#Scaffolds	Gaps (Mb) (% of genome)	Contig N50 (Mb)	Scaffold N50 (Mb)	Genome size (Gb)
10X (NN05)	48370	0.081(4.5)	0.066	42.41	2.06
10X + Bionano (NN05)	48183	0.322 (18)	0.066	147.3	2.11

c

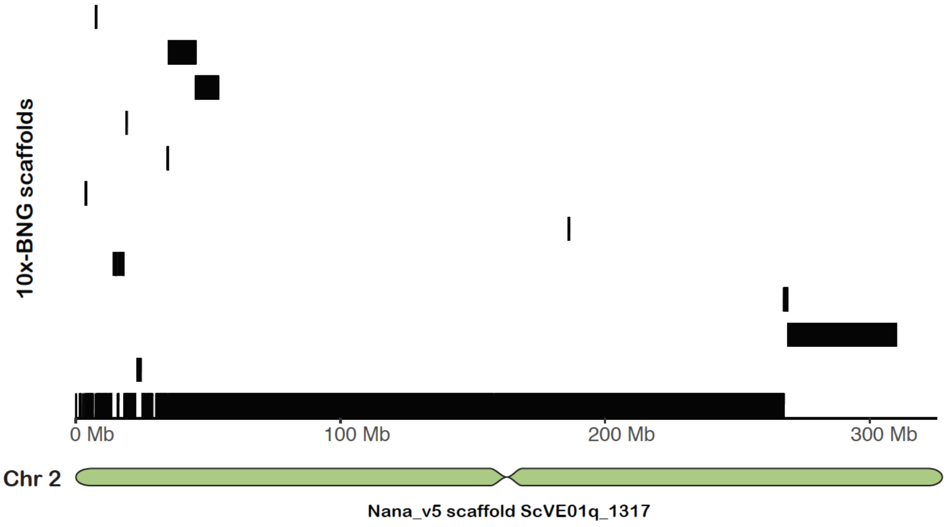

d

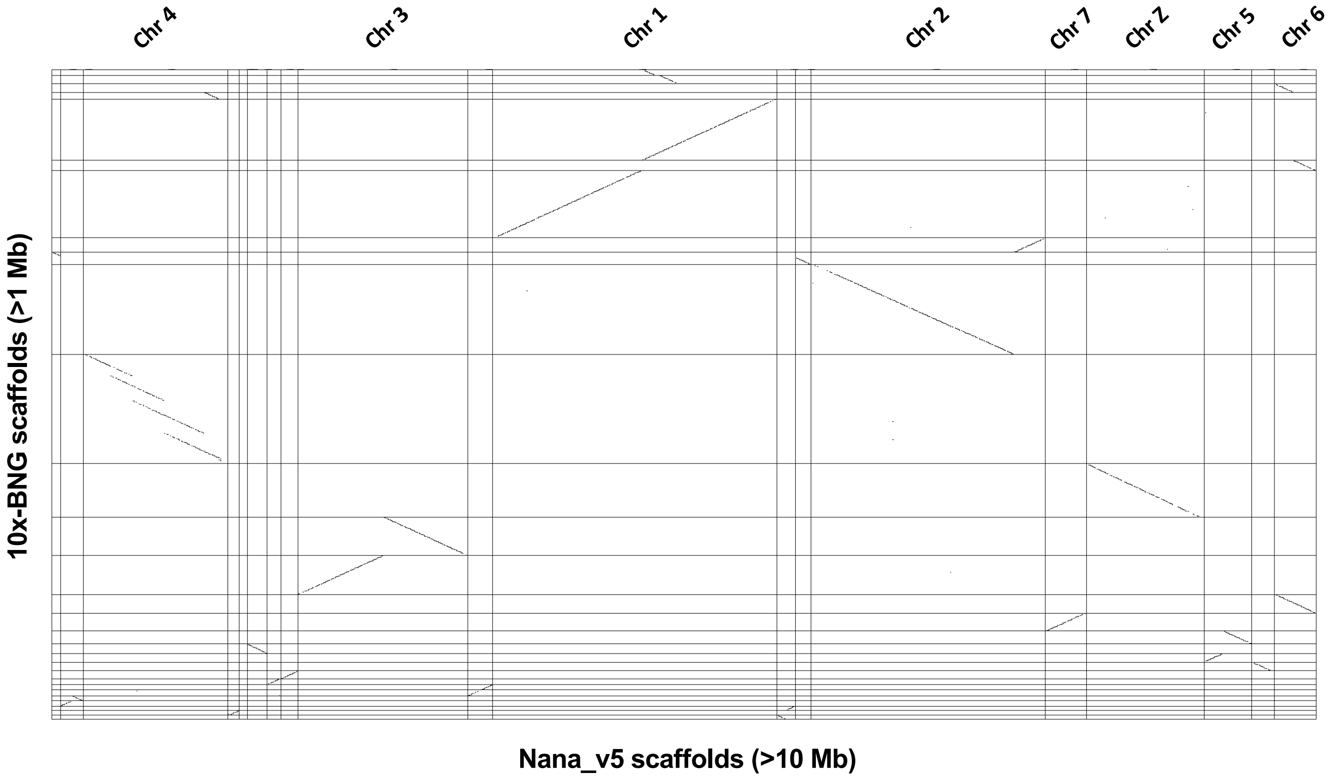

Supplementary Figure 5. 10x genome assembly. (**a**), Schematic of *de novo* assembly using 10x chromium platform. (**b**), Summary statistics of 10x genomics *de novo* genome assembly and 10x-BNG hybrid assembly. (**c**), Alignment of 10x-BNG scaffolds to Nana_v5 chromosome 2 with >90% identity. (**d**), Dot plot alignment of 10x-BNG scaffolds (>1 Mb) and the 19 Nana_v5 scaffolds corresponding to the numbered chromosomes. A female Indian cobra (NN05) was used for 10x *de novo* genome assembly.

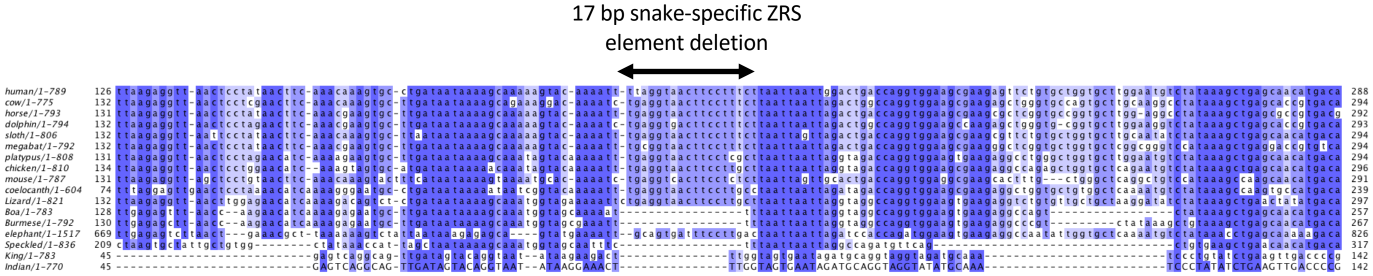

Supplementary Figure 6. Identification of ZRS limb enhancer deletion in Indian cobra genome. Multiple sequence alignment of a core 162 bp mouse ZRS enhancer sequence with orthologous sequences from 16 vertebrate species including *N. naja* generated in this study. The arrow denotes the 17 bp snake-specific deletion that overlaps with a known E1-motif (*Cell*, 2016, **167**:633) required for limb development.

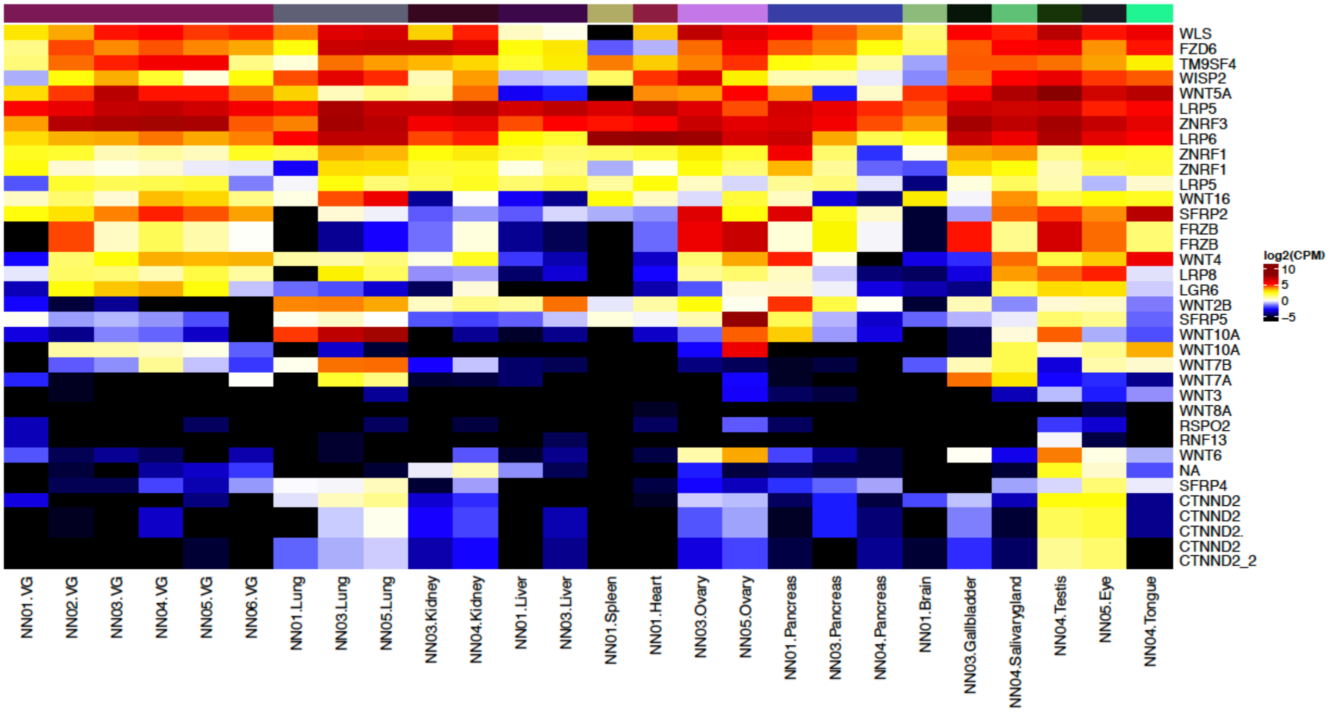

Supplementary Figure 7. Gene expression heatmap of key WNT pathway genes in the Indian cobra. Expression values plotted as log_2 transformed CPM values. NN01 and NN02 correspond to *N. naja* specimens obtained from Kerala, India. NN03, NN04, NN05 and NN06 correspond to *N. naja* specimens obtained from the Kentucky reptile zoo.

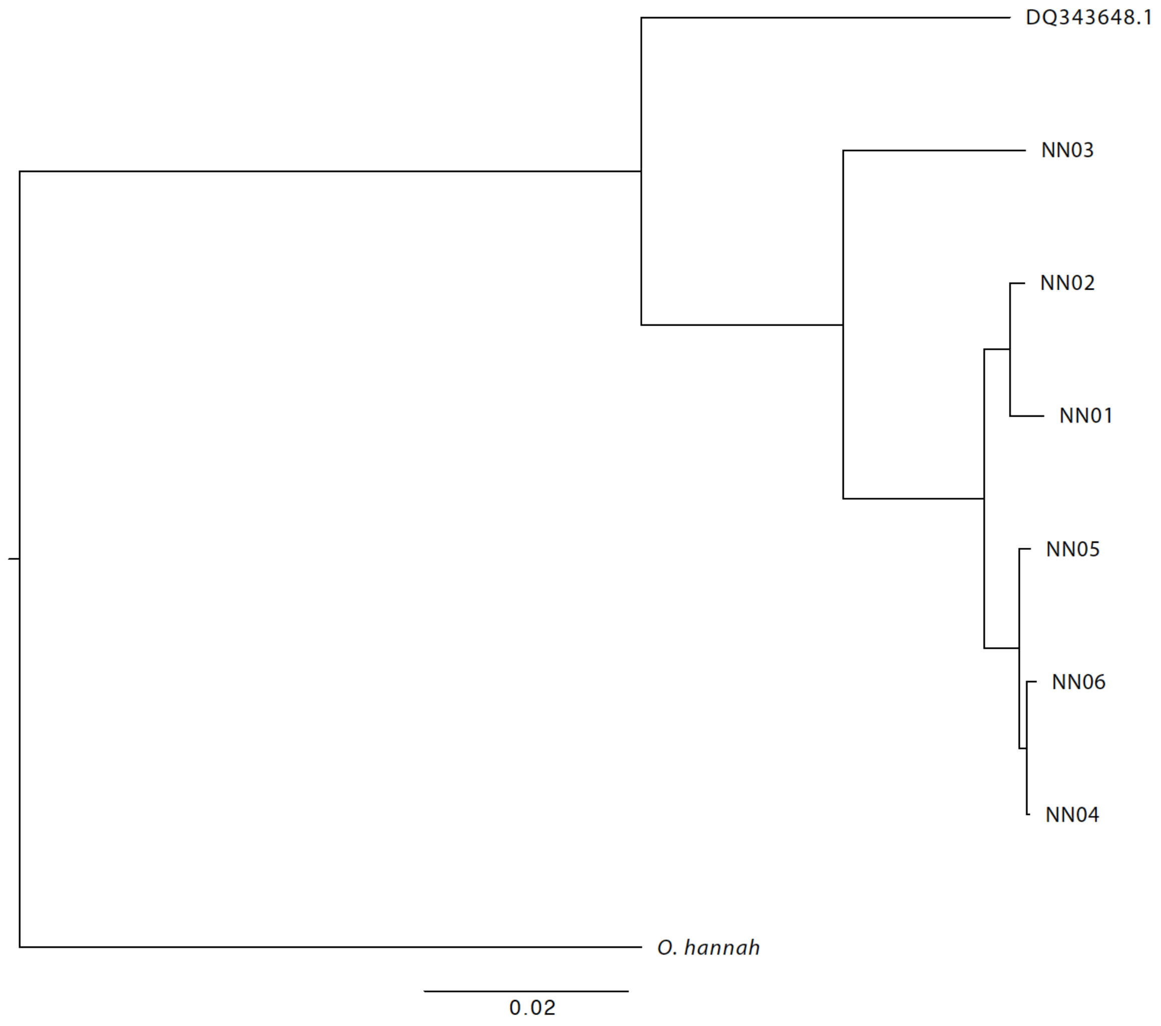

Supplementary Figure 8. Mitochondrial phylogenetic analysis. The bar indicates 0.02 substitutions per nucleotide position. NN01 and NN02 correspond to *N. naja* specimens obtained from Kerala, India. NN03, NN04, NN05 and NN06 correspond to *N. naja* specimens obtained from the Kentucky reptile zoo. GenBank DQ343648.1: *N. naja* published mitochondrial genome. *O. hannah*, *Ophiophagus hannah* (king cobra).

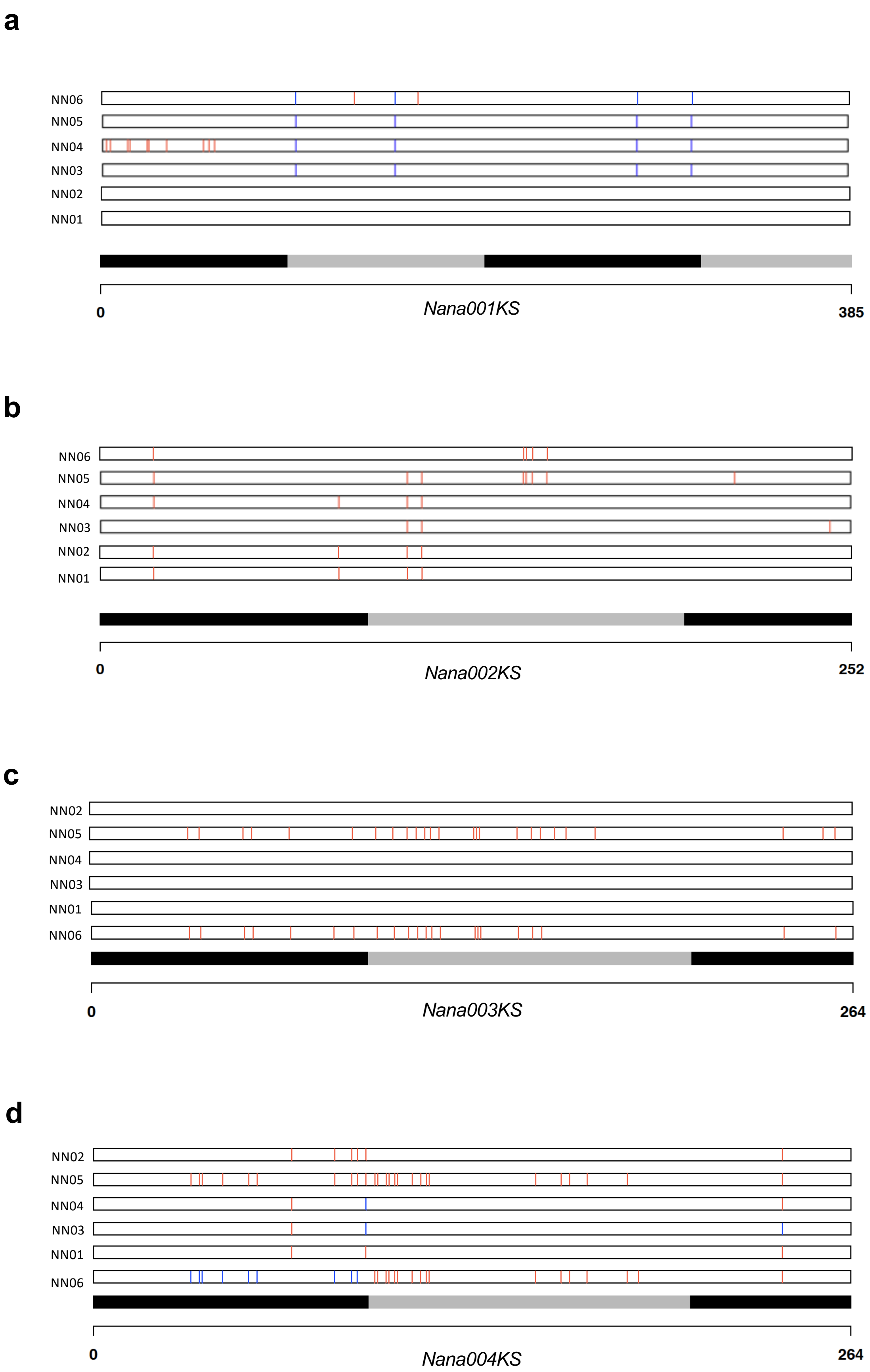
a
NN06
NN05
NN04
NN03
NN02
NN01
0
Nana001KS
385
b
NN06
NN05
NN04
NN03
NN02
NN01
0
Nana002KS
252
c
NN02
NN05
NN04
NN03
NN01
NN06
0
Nana003KS
264
d
NN02
NN05
NN04
NN03
NN01
NN06
0
Nana004KS
264

e

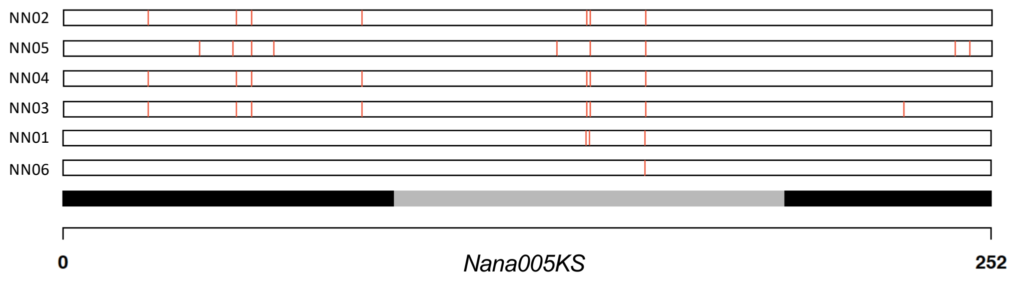
NN02
NN05
NN04
NN03
NN01
NN06
0
Nana005KS
252

f

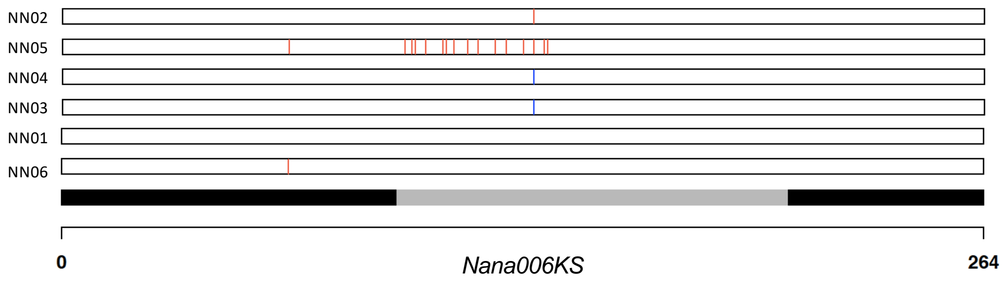
NN02
NN05
NN04
NN03
NN01
NN06
0
Nana006KS
264

g

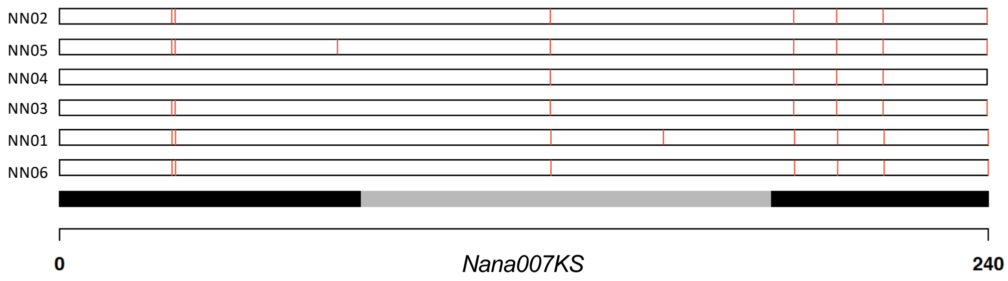
NN02
NN05
NN04
NN03
NN01
NN06
0
Nana007KS
240

h

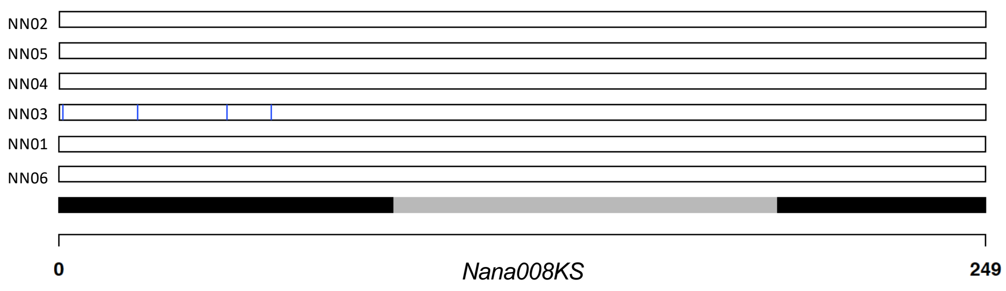
NN02
NN05
NN04
NN03
NN01
NN06
0
Nana008KS
249

i

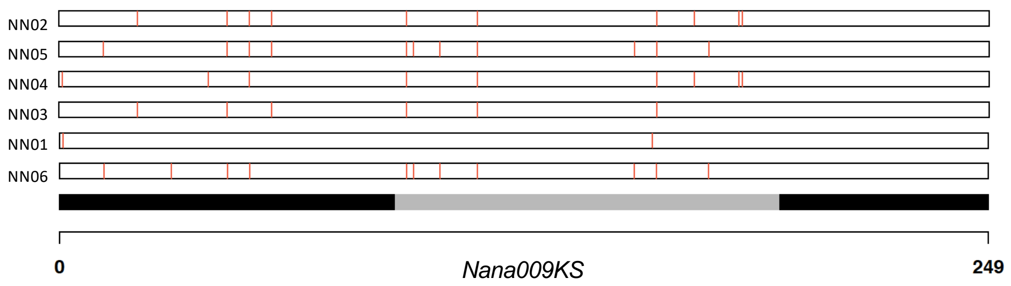
NN02
NN05
NN04
NN03
NN01
NN06
0
Nana009KS
249

j

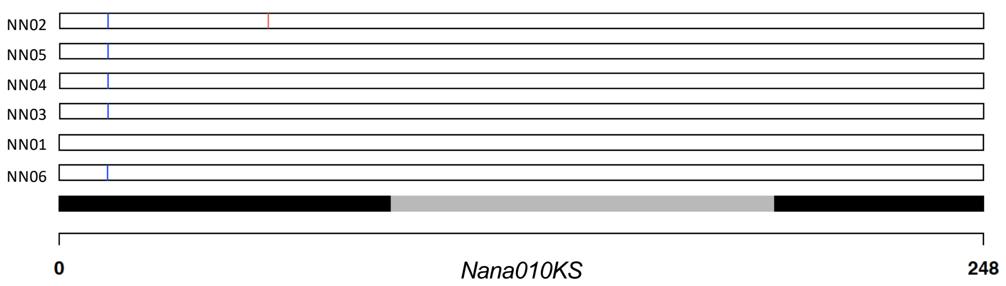
NN02
NN05
NN04
NN03
NN01
NN06
0
Nana010KS
248

k

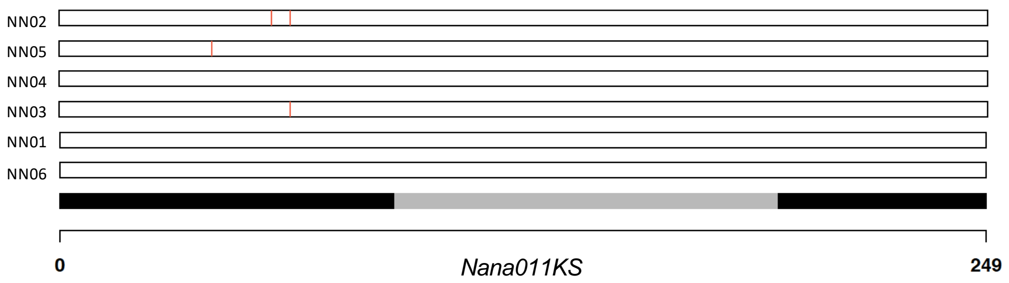
NN02
NN05
NN04
NN03
NN01
NN06
0
Nana011KS
249

l

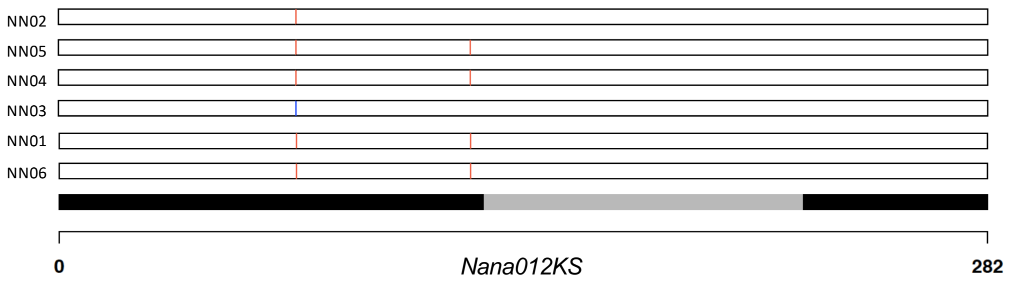
NN02
NN05
NN04
NN03
NN01
NN06
0
Nana012KS
282

m

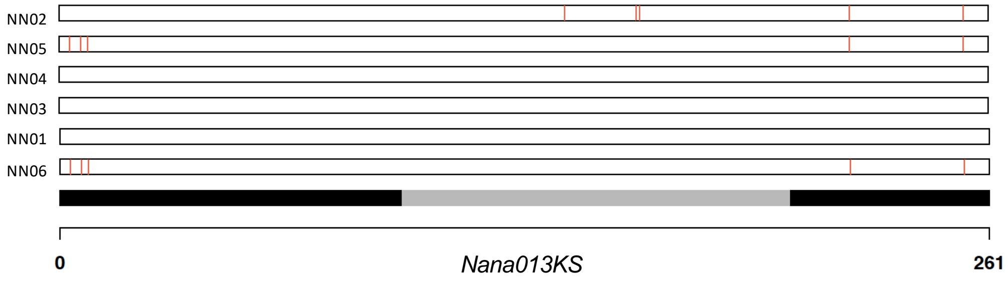
NN02
NN05
NN04
NN03
NN01
NN06
0
Nana013KS
261

n

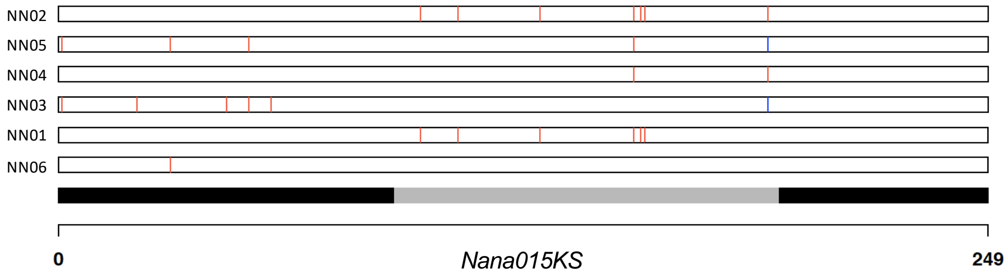
NN02
NN05
NN04
NN03
NN01
NN06
0
Nana015KS
249

o

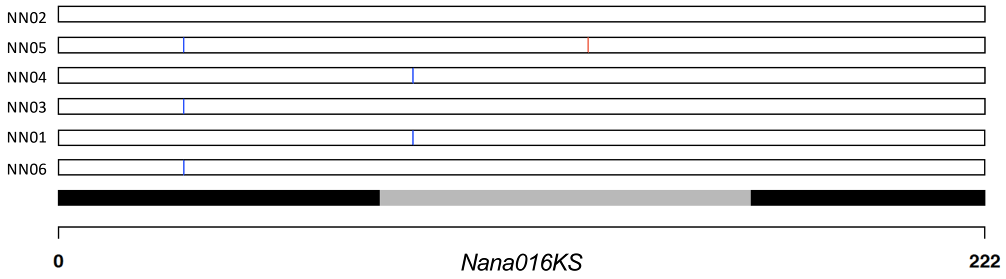
NN02
NN05
NN04
NN03
NN01
NN06
0
Nana016KS
222

p

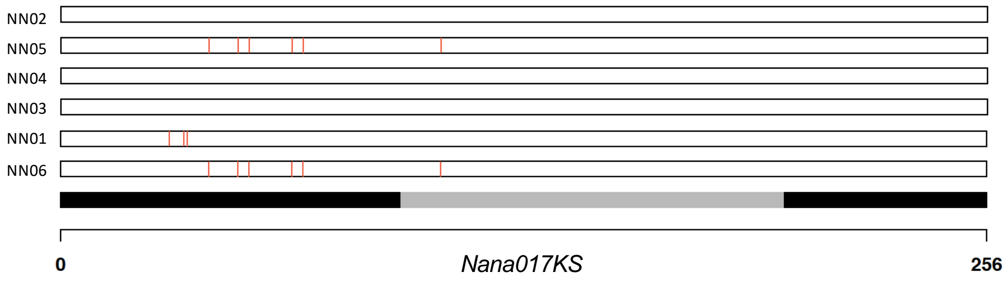
NN02
NN05
NN04
NN03
NN01
NN06
0
Nana017KS
256

q

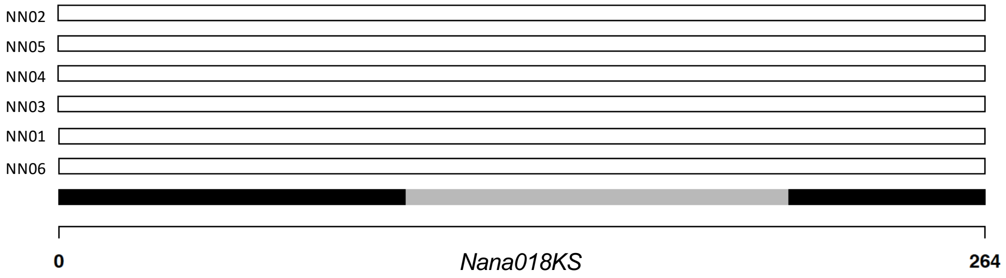

r

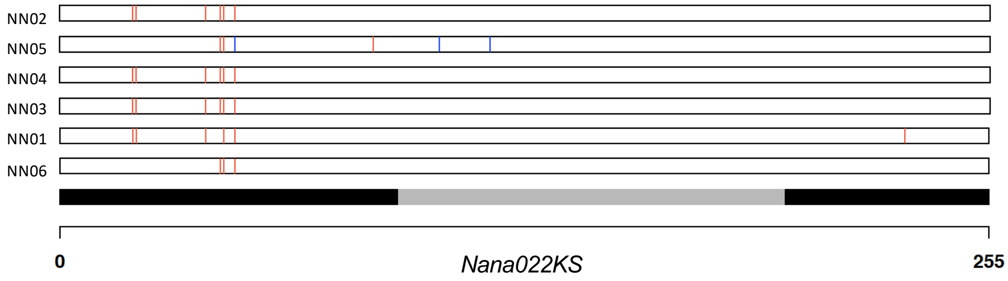

s

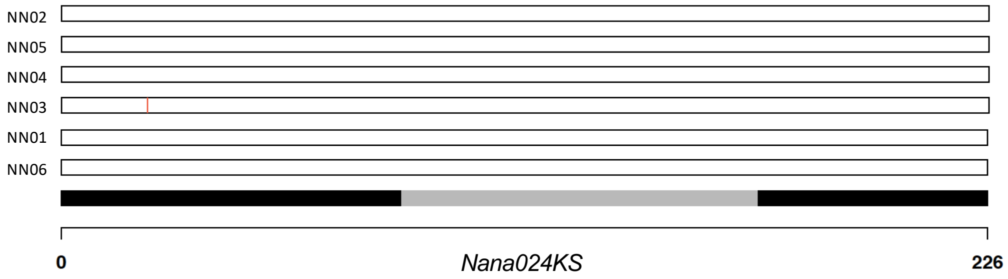

Supplementary Figure 9. Genetic polymorphisms in 6 *N. naja* 3FTx gene family (**a-s**), Protein altering variants in each of the 19 expressed 3FTx genes across all six study animals (NN01-NN06).. Within each track, homozygous variants are shown as blue vertical lines while heterozygous variants are shown as red vertical lines. NN01 and NN02 correspond to *N. naja* specimens obtained from Kerala, India. NN03, NN04, NN05 and NN06 correspond to *N. naja* specimens obtained from the Kentucky reptile zoo.

3. List of Supplementary Tables

Supplementary Table 1a. Sample Information

Supplementary Table 1b. DNA sequencing data generation summary for study animals

Supplementary Table 2a. Summary statistics for scaffolding Nana_v1 with Chicago data

Supplementary Table 2b. Summary statistics for scaffolding Nana_v2 with Bionano data

Supplementary Table 2c. Summary statistics of scaffolding of Nana_v4 with Hi-C data

Supplementary Table 3a. El. quad cDNA marker - Nana_v5 mapping summary

Supplementary Table 3b. Scaffold to Chromosome assignment

Supplementary Table 3c. SChrom-seq mapping data

Supplementary Table 3d. W-chromosome-linked scaffold statistics

Supplementary Table 4a. Indian cobra genome repeat landscape

Supplementary Table 4b. Comparison of repeat elements with other reptile genomes

Supplementary Table 5. Functional annotation summary of predicted Indian cobra protein isoforms

Supplementary Table 6a. Venom gland Pacbio Isoseq data summary

Supplementary Table 6b. Toxin genes annotation

Supplementary Table 6c. Location of toxin gene families in the Indian cobra genome

Supplementary Table 6d. Comparison of venom gene annotations between Indian cobra and prairie rattlesnake

Supplementary Table 6e. Toxin gene orthologs between the Indian cobra and king cobra

Supplementary Table 7a. Number of expressed genes in Indian cobra tissues

Supplementary Table 7b. Predicted protein coding genes in the cobra genome and their expression status

Supplementary Table 7c-h. Tissue-specific differentially upregulated genes

Supplementary Table 8. Peptide sequences from mass spectrometry analysis of pooled Indian cobra venom

Supplementary Table 9. Indian cobra 3FTx protein homology summary

Supplementary Table 10. Structural modeling of representative Indian cobra 3FTx proteins

Supplementary Table 11. Gene pathway enrichment analysis of Indian cobra genes

Supplementary Table 12a: Pairwise genotype similarity analysis using protein altering variants

Supplementary Table 12b. Protein altering variants in venom gland genes

Supplementary Table 12c. Protein altering variants in expressed venom gland-specific genes

Supplementary Table 12d. Protein altering variants in 3FTx genes
