# Supplementary material for: The Indian cobra reference genome and transcriptome enables comprehensive identification of venom toxins
Source: Nat Genet. 2020 Jan 6;52(1):106–17. doi: 10.1038/s41588-019-0559-8 (PMC8075977; doi:10.1038/s41588-019-0559-8)
Supplement: Supplementary file 2 — Reporting Summary [file 41588_2019_559_MOESM2_ESM.pdf]

natureresearch

Corresponding author(s): Somasekar Seshagiri

Last updated by author(s): Nov 19, 2019

Reporting Summary

Nature Research wishes to improve the reproducibility of the work that we publish. This form provides structure for consistency and transparency in reporting. For further information on Nature Research policies, see Authors & Referees and the Editorial Policy Checklist.

Statistics

For all statistical analyses, confirm that the following items are present in the figure legend, table legend, main text, or Methods section.

n/a	Confirmed	
☐	☒	The exact sample size (*n*) for each experimental group/condition, given as a discrete number and unit of measurement
☐	☒	A statement on whether measurements were taken from distinct samples or whether the same sample was measured repeatedly
☐	☒	The statistical test(s) used AND whether they are one- or two-sided<br>*Only common tests should be described solely by name; describe more complex techniques in the Methods section.*
☒	☐	A description of all covariates tested
☐	☒	A description of any assumptions or corrections, such as tests of normality and adjustment for multiple comparisons
☐	☒	A full description of the statistical parameters including central tendency (e.g. means) or other basic estimates (e.g. regression coefficient) AND variation (e.g. standard deviation) or associated estimates of uncertainty (e.g. confidence intervals)
☐	☒	For null hypothesis testing, the test statistic (e.g. *F*, *t*, *r*) with confidence intervals, effect sizes, degrees of freedom and *P* value noted<br>*Give P values as exact values whenever suitable.*
☒	☐	For Bayesian analysis, information on the choice of priors and Markov chain Monte Carlo settings
☒	☐	For hierarchical and complex designs, identification of the appropriate level for tests and full reporting of outcomes
☒	☐	Estimates of effect sizes (e.g. Cohen's *d*, Pearson's *r*), indicating how they were calculated

Our web collection on statistics for biologists contains articles on many of the points above.

Software and code

Policy information about availability of computer code

Data collection	No commercial/custom code was used for data collection.
Data analysis	We list here the software URLs of all open-source tools used for data analysis.<br>R: https://www.R-project.org/<br>Arrow: https://github.com/PacificBiosciences/GenomicConsensus<br>Canu: https://github.com/marbl/canu<br>Flye: https://github.com/fenderglass/Flye<br>WTDBG https://github.com/ruanjue/wtdbg2<br>BUSCO: https://gitlab.com/ezlab/busco<br>Jellyfish: https://www.cbcb.umd.edu/software/jellyfish/<br>GenomeScope: http://qb.cshl.edu/genomescope/<br>Repeatmasker: http://www.repeatmasker.org<br>Repeatmodeler: http://www.repeatmasker.org/RepeatModeler.html<br>MAKER: http://www.yandell-lab.org/software/maker.html<br>SNAP: http://snap.cs.berkeley.edu<br>AUGUSTUS: http://augustus.gobics.de/<br>Exonerate: https://www.ebi.ac.uk/about/vertebrate-genomics/software/exonerate<br>snpEff: http://snpeff.sourceforge.net/<br>Enrichr: http://amp.pharm.mssm.edu/Enrichr<br>GATK: https://software.broadinstitute.org/gatk/download/<br>BWA: https://github.com/lh3/bwa/releases<br>EdgeR: https://bioconductor.org/packages/release/bioc/html/edgeR.html<br>Synteny plots: https://github.com/biopython/biopython/blob/master/Doc/examples/Proux_et_al_2002_Figure_6.py<br>OrthoFinder: http://www.stevekellylab.com/software/orthofinder<br>STAR: https://github.com/alexdobin/STAR

featureCounts: http://bioinf.wehi.edu.au/featureCounts/
Picard: http://broadinstitute.github.io/picard/
Figtree: https://github.com/rambaut/figtree
CoGe: https://genomevolution.org/coge/
Symap: http://www.agcol.arizona.edu/software/symap/
vt: https://github.com/atks/vt

For manuscripts utilizing custom algorithms or software that are central to the research but not yet described in published literature, software must be made available to editors/reviewers. We strongly encourage code deposition in a community repository (e.g. GitHub). See the Nature Research guidelines for submitting code & software for further information.

Data

Policy information about availability of data

All manuscripts must include a data availability statement. This statement should provide the following information, where applicable:

- Accession codes, unique identifiers, or web links for publicly available datasets
- A list of figures that have associated raw data
- A description of any restrictions on data availability

All sequencing data that support this study have been deposited in NCBI under the BioProject accession # PRJNA527614 and can be accessed at "https://dataview.ncbi.nlm.nih.gov/object/PRJNA527614".
Mass spectrometry data collected in this study has been deposited at MassIVE and can be accessed via this link - "ftp://massive.ucsd.edu/MSV000084564/".

Field-specific reporting

Please select the one below that is the best fit for your research. If you are not sure, read the appropriate sections before making your selection.

☒ Life sciences ☐ Behavioural & social sciences ☐ Ecological, evolutionary & environmental sciences

For a reference copy of the document with all sections, see nature.com/documents/nr-reporting-summary-flat.pdf

Life sciences study design

All studies must disclose on these points even when the disclosure is negative.

Sample size	A total of 6 animals was used in this study as described in Table S1a and methods section.
Data exclusions	No data was excluded in this study.
Replication	Biological replicates, where appropriate, have been used and described in the methods section and supplementary tables
Randomization	This is a de novo genome sequencing project and hence, randomization does not apply to this study.
Blinding	This is a de novo genome sequencing project and hence, blinding does not apply to this study.

Reporting for specific materials, systems and methods

We require information from authors about some types of materials, experimental systems and methods used in many studies. Here, indicate whether each material, system or method listed is relevant to your study. If you are not sure if a list item applies to your research, read the appropriate section before selecting a response.

Materials & experimental systems

n/a	Involved in the study	
☒	☐	Antibodies
☒	☐	Eukaryotic cell lines
☒	☐	Palaeontology
☐	☒	Animals and other organisms
☒	☐	Human research participants
☒	☐	Clinical data

Methods

n/a	Involved in the study	
☒	☐	ChIP-seq
☐	☒	Flow cytometry
☒	☐	MRI-based neuroimaging

Animals and other organisms

Policy information about studies involving animals; ARRIVE guidelines recommended for reporting animal research

Laboratory animals	We obtained horse blood from a horse (Equus caballus) that is a research mare at the Texas A&M Large Animal clinic.<br>Species: Equus caballus<br>Breed: American quarter horse

	Sex: female<br>Age: 6 years old
Wild animals	Two animals used in this study from India were fresh road kills and were approved for use in the study as described in the methods section.
Field-collected samples	This study did not include field-collected samples
Ethics oversight	This study did not require ethics approval.

Note that full information on the approval of the study protocol must also be provided in the manuscript.

Flow Cytometry

Plots

Confirm that:

- [x] The axis labels state the marker and fluorochrome used (e.g. CD4-FITC).
- [x] The axis scales are clearly visible. Include numbers along axes only for bottom left plot of group (a 'group' is an analysis of identical markers).
- [ ] All plots are contour plots with outliers or pseudocolor plots.
- [x] A numerical value for number of cells or percentage (with statistics) is provided.

Methodology

Sample preparation	Sample preparation and processing are described in the materials and methods section of the main text.
Instrument	BD Accuri™ C6 personal flow cytometer
Software	BD Accuri C6
Cell population abundance	Described in materials and methods section of main text
Gating strategy	Gating strategy is provided in Supplementary Fig. 1a

- [x] Tick this box to confirm that a figure exemplifying the gating strategy is provided in the Supplementary Information.
